# Battling Enteropathogenic Clostridia: Phage Therapy for *Clostridioides difficile* and *Clostridium perfringens*

**DOI:** 10.3389/fmicb.2022.891790

**Published:** 2022-06-13

**Authors:** Jennifer Venhorst, Jos M. B. M. van der Vossen, Valeria Agamennone

**Affiliations:** ^1^Biomedical Health, Netherlands Organisation for Applied Scientific Research (TNO), Utrecht, Netherlands; ^2^Microbiology and Systems Biology, Netherlands Organisation for Applied Scientific Research (TNO), Zeist, Netherlands

**Keywords:** phage (bacteriophage), endolysin, enteropathogen, *Clostridioides difficile*, *Clostridium perfringens*, phage therapy

## Abstract

The pathogenic *Clostridioides difficile* and *Clostridium perfringens* are responsible for many health care-associated infections as well as systemic and enteric diseases. Therefore, they represent a major health threat to both humans and animals. Concerns regarding increasing antibiotic resistance (related to *C. difficile* and *C. perfringens*) have caused a surge in the pursual of novel strategies that effectively combat pathogenic infections, including those caused by both pathogenic species. The ban on antibiotic growth promoters in the poultry industry has added to the urgency of finding novel antimicrobial therapeutics for *C. perfringens*. These efforts have resulted in various therapeutics, of which bacteriophages (in short, phages) show much promise, as evidenced by the Eliava Phage Therapy Center in Tbilisi, Georgia (https://eptc.ge/). Bacteriophages are a type of virus that infect bacteria. In this review, the (clinical) impact of clostridium infections in intestinal diseases is recapitulated, followed by an analysis of the current knowledge and applicability of bacteriophages and phage-derived endolysins in this disease indication. Limitations of phage and phage endolysin therapy were identified and require considerations. These include phage stability in the gastrointestinal tract, influence on gut microbiota structure/function, phage resistance development, limited host range for specific pathogenic strains, phage involvement in horizontal gene transfer, and—for phage endolysins—endolysin resistance, -safety, and -immunogenicity. Methods to optimize features of these therapeutic modalities, such as mutagenesis and fusion proteins, are also addressed. The future success of phage and endolysin therapies require reliable clinical trial data for phage(-derived) products. Meanwhile, additional research efforts are essential to expand the potential of exploiting phages and their endolysins for mitigating the severe diseases caused by *C. difficile* and *C. perfringens*.

## Introduction

Although species of *Clostridium* cluster XIVa and IV, designated by 16S rRNA phylogeny and identified as the predominant bacteria in the gut, are indispensable for intestinal homeostasis (Guo et al., 2020), pathogenic species belonging to the class of Clostridia represent a major health threat to both humans and animals. Among them, two bacterial species have a disreputable reputation of being involved in severe gastrointestinal diseases: *Clostridioides difficile* [Cd; formerly *Clostridium difficile* (Lawson et al., 2016)] and *Clostridium perfringens* (Cp). Cd has been recognized as the most common pathogen causing health care-associated infections, accounting for 15% of all such infections in the United States (Magill et al., 2018) and presenting a major burden on health care systems (Marra et al., 2020). Cp, on the other hand, has been implicated in significant systemic and enteric diseases, including gas gangrene (Clostridial myonecrosis), food poisoning and non-foodborne diarrhea, enterocolitis in both humans and animals (Kiu and Hall, 2018), and necrotizing enterocolitis (NEC) in preterm infants. This pathogen is furthermore responsible for an estimated $6 billion in annual losses to the global poultry industry by inducing necrotic enteritis, an enteric disease characterized by patches of necrotic tissue on intestinal epithelium, in chicks (Wade and Keyburn, 2015).

Due to the large societal and economic impact of Cp and Cd infections, and concerns regarding increasing antibiotic resistance related to Cp (Xiaoting et al., 2021) and Cd (Imwattana et al., 2019), effective treatment and prevention strategies are in high demand. Among those, the use of bacteriophages (phage therapy) and phage-derived endolysins to treat bacterial infections has attracted recent attention. This review will revisit the characteristics of Cp and Cd and phages in general, followed by a description of the state-of-art concerning the application and engineering of bacteriophages and their endolysins in battling infections caused by Cp and Cd. Finally, challenges, limitations and future perspectives concerning the (clinical) use of phage therapy against these intestinal disease-causing enteropathogens will be discussed.

### Clostridium perfringens

The anaerobic, rod-shaped, Gram-positive bacterium *C. perfringens* (Cp), belonging to the Clostridiaceae family, resides in the top six of bacteria causing food poisoning in the United States (Scallan et al., 2015). It is widely distributed in the intestines of animals, especially poultry, as well as in diverse other environments. This is in part due to its ability to form spores that are highly resilient to stressors, such as toxic chemicals, heat and radiation (Paredes-Sabja et al., 2008). Cp is a versatile pathogen of humans and husbandry animals causing wound infections, enteritis/enterocolitis and enterotoxaemia (for recent review see Mehdizadeh Gohari et al., 2021).

### Virulence

The virulence of Cp can largely be ascribed to its vast arsenal of toxins. These toxins are used to classify isolates to one of seven toxin types (A–G) (Rood et al., 2018). Specific toxin types, in turn, correspond with different disease manifestations. Toxin type F (producing alpha toxin and *C. perfringens* enterotoxin), for example, is associated with human food poisoning, antibiotic-associated diarrhea, and sporadic diarrhea. Type G [producing alpha toxin and Necrotic enterocolitis B-like toxin (NetB)] is linked to necrotic enteritis of poultry (Cooper et al., 2013; Rood et al., 2018; Mehdizadeh Gohari et al., 2021). In addition to NetB, a recent genomic analysis positively associated the toxin gene *tpe*L, and other virulence genes *pfo*A, *cpb*2 and *cna* to necrotic enteritis-linked isolates (Kiu et al., 2019). Lepp et al. (2021) furthermore showed the important role of adhesive pilus of toxin type G strains in causing necrotic enteritis in poultry. Toxin type C causes severe and lethal necrotic enteritis in newborn piglets (Posthaus et al., 2020).

Cp strains st13 and SM101 have fully sequenced genomes and have been the most extensively studied with respect to *C. perfringens* pathogenicity (Sekiya et al., 2021a). Aside from these fully sequenced strains, further comparative genome analysis of 206 publicly available Cp strains’ sequence data indicated five closely-related phylogenetic groups (phylogroups) reflecting different disease involvements (Abdel-Glil et al., 2021), thus demonstrating the large repertoire of potential virulence factors. Particularly phylogroup 1 contained the human food poisoning strains producing enterotoxin and the Darmbrand strain. Kiu and Hall (2018) schematically depicted the disease-linked virulence factors of Cp in the context of intestinal infections. They also reviewed the capacity of abundant histotoxic gas production alongside rapid growth of Cp known as clostridial myonecrosis (gas gangrene). Excessive hydrogen sulfide produced by Cp is toxic to human cells and is associated with intestinal inflammatory diseases such as ulcerative colitis (Rowan et al., 2009) and NEC in preterm infants with severe intestinal tissue necrosis and gas production (Neu and Walker, 2011).

### Treatment and Preventive Strategies

Infections with Cp cause severe disease in extremely low birth weight preterm infants, with an incidence that varies from 2 to 13% (Alsaied et al., 2020) and a mortality rate of 30–50% with surgical NEC (Kiu and Hall, 2018). To prevent NEC in preterm infants various strategies have been explored, including the introduction of probiotics (Alfaleh and Anabrees, 2014), breast milk feeding and probiotics (Jin et al., 2019) and human milk oligosaccharides to support bifidobacteria and lactic acid bacteria (Bode, 2018). Phage introduction *via* fecal transplantation was discussed as a solution (Hofer, 2021). Despite safety concerns related to the possible unintended transfer of pathogens *via* fecal transplantation, phage therapy *via* this route has been suggested as a good option to explore (Hofer, 2021).

Since the European ban in 2006 on the use of antibiotics as growth promotors in animal feed, alternative strategies to reduce diseases in animals have been explored also to ensure food safety. The preventive strategies against necrotic enteritis in poultry, caused by Cp, has gained a lot of attention. In the poultry industry, natural feed additives were shown effective in reducing or even abolishing Cp infections (Caly et al., 2015). Typical naturals explored were live bacteria and yeasts (probiotics), plants and plant molecules (such as essential oils), organic acids, enzymes, lysozymes, antimicrobial peptides, bacteriocins (i.e., antimicrobial peptides produced by bacteria), vaccines and bacteriophages. Antimicrobial molecules, such as bacteriocins or phage endolysins, were considered good candidates for new antimicrobials (Caly et al., 2015).

### Associated Societal Costs

In addition to the health burden delineated above, Cp infections are accompanied with extremely high costs. The care for NEC-affected infants amounts to $500 million to $1 billion in the United States (Neu and Walker, 2011). The financial loss for the poultry sector has been estimated close to $6 billion in the United States in 2015 (Wade and Keyburn, 2015).

## Clostridioides difficile

*Clostridioides difficile* [Cd; formerly *Clostridium difficile* (Lawson et al., 2016)] is an anaerobic, rod-shaped, Gram-positive bacterium belonging to the Peptostreptococcaceae family. It displays important virulence factors, such as sporulation, toxin production, and mucosal biofilm formation (Lawson et al., 2016). Cd is mainly active in elderly upon use of antibiotics that negatively affect the colonic microbiome, thereby providing space for overgrowth of the bacterium. Its toxins cause severe colitis. As Cd’s spores are highly resistant to environmental stress, the bacterium can easily spread among the population in aged care facilities (for review see Czepiel et al., 2019). Moreover, next generation sequencing on a large number of Cd genomes indicated that horizontal gene transfer of mobile genetic elements (conjugative transposons and prophages) likely explains the plasticity of the *C. difficile* genome and its rapid change in epidemiology (Meessen-Pinard et al., 2012; Knight et al., 2015).

### Virulence

*Clostridioides difficile* encodes genetic variants of three well characterized toxins at two distinct loci: toxin A and B in the pathogenicity locus (PaLoc) and Cd binary toxin in the correspondingly named locus (CdtLoc). While the former two toxins represent primary virulence factors for Cd infection, Cd binary toxin has been associated with increased severity of Cd infection. Cd binary toxin-induced actin depolymerization produces microtubule-based membrane protrusions, which form a network on epithelial cells and increase bacterial adherence (Gerding et al., 2013). The production of the three toxins is primarily controlled by regulators specific to each locus (Majumdar and Govind, 2022), and has been described to be strain-specific. For example, the regulator of Cd binary toxin production (CtdR) increased the production of toxin A, toxin B and Cd binary toxin in two epidemic ribotype 027 human strains, but not in a ribotype 078 animal strain (Lyon et al., 2016). Toxin A and toxin B production in the PaLoc can be influenced by some prophages, e.g., by phiCD38-2 (Supplementary Table 2). PaLoc has some features in common with phages. In particular *tcd*E, a gene encoding a membrane protein similar to a phage holin, was shown to promote toxin secretion. This observation shows the linkage between phages and Cd virulence (Fortier, 2017).

Cd can be classified based on ribotype (Lawson et al., 2016) but other classification schemes have been proposed as well. Based on the fact that neither ribotyping nor sequence typing could predict the toxin genotype of 478 whole genome sequenced isolates, it was proposed to also include toxin variant type for comprehensive Cd strain classification (Li Z. et al., 2020).

*Clostridioides difficile* was first recognized as a pathogen in 1978 (Gerding, 2009), and, soon thereafter, Cd infection (CDI) became the most frequently observed cause of antibiotic-associated diarrhea in hospitals, causing 15–25% of all cases (Bartlett and Gerding, 2008). Toxigenic strains are part of the infant microbiota, and indeed Cd is frequently encountered in the gut microbiota of infants without any symptoms of disease (Adlerberth et al., 2014). However, disruption of microbiota composition by malnutrition or antibiotic use can initiate CDI with increasing Cd toxin concentrations, Cd colonization and biofilm formation in the gut, resulting in a severe disease status (Vedantam et al., 2012). Especially broad-spectrum antibiotics, such as ampicillin, clindamycin and third-generation cephalosporins, are considered important risk factors for CDI (Oldfield et al., 2014). This millennium, more epidemic types of Cd have increased the incidence of CDI and changed epidemiology and severity of disease particularly for elderly in hospital and nursing homes. Those more epidemic types are represented by ribotype 027 (Denève et al., 2009) and ribotype 078 (Goorhuis et al., 2008). Dietary trehalose further enhances virulence of epidemic Cd strains (Collins et al., 2018). On top of this, Cd is now increasingly community acquired (Smits et al., 2016). An important reason for this is the fact that Cd has an extensive repertoire of properties to survive harsh conditions. Spores of this bacterium can survive high temperature, disinfectants, and antibiotics, thereby promoting the spreading of this bacterium. This phenomenon, combined with a compromised immune system of elderly and hospitalized patients, explains their role in nosocomial infections upon antibiotic treatment. Cd colonization can be observed in up to 73% in inpatients above 65 years old (Denève et al., 2009).

### Treatment and Preventive Strategies

Standard treatment of patients with mild CDI relies on withdrawing broad spectrum antibiotics and correcting fluid loss due to the antibiotic-associated diarrhea. Treatment of severe and recurrent CDI currently relies on the use of antibiotics vancomycin and fidaxomicin (Debast et al., 2014). Also fecal microbiota transplantation, first used in the 1950s, has become an established treatment option (Meighani et al., 2017). Fecal microbiota transplantation appeared successful with a cure rate of approximately 93% in patients with recurrent CDI and was therefore accepted as a valid alternative to failing antibiotic treatment (Kassam et al., 2012). Despite the somewhat successful treatments currently used, novel therapeutic strategies are urgently needed.

Preventive strategies for CDI are more important than curing and could be established *via* reduction of patient susceptibility, prevention of transmission of Cd in health care institutions and rapid diagnosis of Cd patients (Surawicz et al., 2013). Antimicrobial stewardship is also highly recommended in view of prevention. Other options for prevention are being explored, including vaccines that are currently under clinical trials (Henderson et al., 2017; Legenza et al., 2017). A number of studies showed the potential benefit of probiotics in preventing CDI upon timely use in hospitalized adults receiving antibiotics, although the efficacy of probiotics was reduced by half when probiotics were administered more than 2 days after antibiotic treatment was started (Shen et al., 2017). A new potentially preventive measure studied *in vitro* was the use of the human milk oligosaccharides 2′-*O*-fucosyllactose and lacto-N-neotetraose. These human milk oligosaccharides or their mixture decreased the presence of Cd in human gut microbiota versus untreated conditions. This decrease coincided with increased acetate and Bifidobacteriaceae levels, thereby suggesting a microbiota-modulated anti-Cd effect, rather than a direct effect of the human milk oligosaccharides (Vigsnaes et al., 2021). These findings suggest that CDI can possibly be prevented by modulating microbiota composition and activity.

### Associated Societal Costs

An issue in the treatment of CDI is the frequency of recurrence. This is observed in about 15–30% of the cases, making CDI an important clinical and life-threatening issue (Song and Kim, 2019). As second recurrence rates increased to about 40 and to 65% for next recurrences, recurrent CDI is very difficult to treat and health care costs are dramatically increased by CDI patients (Ghantoji et al., 2010). Primary and recurrent CDI were calculated to cost up to $5,000 and $18,000 per case, respectively. Ghantoji et al. (2010) suggested therefore that the enormous increase in the occurrence of CDI with impressive numbers of patients and related high costs for health-care justifies an investment in CDI prevention and control.

In the presence of an already established *Clostridioides* infection, when antibiotics have proven ineffective (for example because of the emergence of resistance, or because of the risk of side-effects that may exacerbate the disease phenotype), alternative therapeutic options need to be explored. Bacteriophages represent a possible solution in this scenario (Zuo et al., 2018).

## Bacteriophages and Their Emerging Use as Therapeutic Agents

Bacteriophages are viruses that infect bacteria, showing bactericidal effects against both Gram-positive and Gram-negative bacteria. Phages are highly diverse in shape and size (White and Orlova, 2019). Nevertheless, tailed bacteriophages are classified as a single order of Caudovirales based on similarities in the virionic structure, genome organization and genetic material expression (Kwiatek et al., 2020). Caudovirales are the most studied group and are thought to account for 96% of all phages (Zinke et al., 2022). The tailed phages share a common structural organization, including a capsid containing a densely packed double-stranded DNA and a tail. Depending on the morphology of the tail, tailed phages can be further classified to the Myoviridae, Siphoviridae, and Podoviridae families (Zinke et al., 2022). The function of the phage tail is to recognize and attach to the host cells by means of receptor binding proteins (RBPs), to subsequently penetrate the cell envelope barrier, and ultimately to deliver DNA into the cytoplasm. Tail complexes vary both in numbers and types of RBPs. Combined with the various types of receptors on the host surface, bacteriophages likely use a diverse range of structural modules for host-cell recognition that ultimately govern host specificity (Huang and Xiang, 2020).

After infection, phages can follow one of two possible life cycles: the lytic or lysogenic cycle (Hobbs and Abedon, 2016). During the lysogenic cycle, the virion DNA is incorporated in the host genome. The resulting prophage, i.e., the integrated phage DNA, replicates with the host cell without damaging it, until the lytic cycle is triggered. Such triggers include environmental factors, for example, UV radiation or exposure to toxic chemicals. During the lytic cycle, the phage uses the bacterial cellular machinery to produce phage progeny and phage lytic enzymes, that break the bacterial cell wall to ensure phage release (Jamal et al., 2019). Among the lytic enzymes are endolysins, which are secreted at the end of the phage lytic cycle and hydrolyze the host cell wall from within. Endolysins are also capable of degrading the host peptidoglycan externally without the assistance of the intact phage; these entities represent anti-microbial therapeutics in their own right (Abdelrahman et al., 2021).

Even though the lytic activity of phages and their encoded lytic enzymes were already discovered in the second decade of the 1900s (Twort, 1915), their therapeutic use re-gained interest with the emergence of antibiotic resistance (Ho, 2001). Since then, it has become clear that phage therapy and the application of its endolysins offer not only a potential solution to the problem of antibiotic resistance, but also the possibility to apply more specific and targeted antibacterial treatments (Kwiatek et al., 2020). This is due to their narrow host range, i.e., the bacterial genera, species and strains they can lyse (Kutter, 2009). As such, bacteriophages and phage-encoded endolysins circumvent the dysbiosis and subsequent overgrowth of pathogenic species often associated with antibiotic treatment (Chan et al., 2013). Phages and endolysins thus offer advantages over antibiotics with potential use in human and veterinary medicine, but also dentistry, agriculture, and biocontrol. In 2019, the first clinical trial for intravenous phage therapy was approved by the US Food and Drug Administration for the treatment of resistant *Staphylococcus aureus* infections (Petrovic Fabijan et al., 2020), underlining the therapeutic potential of, and high medical need for, phages as curative antimicrobial treatment. Nevertheless, their application—and that of endolysins—also come with challenges and risks that can only be overcome by a thorough understanding of their *in vivo* physicochemical properties and behavior, the mechanisms of action pertaining to both host specificity and infection potential and their safety liabilities.

## Bacteriophages and Phage-Derived Endolysins Against *Clostridium perfringens*

### Phages Against *Clostridium perfringens*

Phages of *C. perfringens* belong to the order of Caudovirales. Table 1 lists the Cp phages described to date in the literature. The best characterized phages belong to the Podoviridae family and Picovirianae subfamily, and the majority of Cp phages originate from poultry. All phages described here selectively target Cp, with no known activity toward other bacteria. Nevertheless, differences in host range of the phages have been observed within the Cp species. For example, phage 3626 was lytic against 11 out of 51 strains examined (21.6%), while phage 8533 was able to lyse only four strains (7.8%) which overlapped with phage 3626 sensitive strains (Zimmer et al., 2002a). The high specificity of phages provides potential therapeutic advantages. By only targeting the infection-causing pathogen, the gut microbiome—necessary for gut homeostasis—may remain intact. Phage engineering, focusing either on optimal physicochemical properties or host range, may further enhance the applicability of phages and enable therapeutic application as described below.

**TABLE 1:** *Clostridium perfringens* phages characterized to date, as described in the referenced literature.

Phage	Family (Subfamily)	Growth cycle	Origin	Host (Toxinotype)	Sensitive Cp strains (Toxinotype)	Study references	Genbank accession number	Main findings of the study
Susfortuna	Podoviridae (Picovirinae)	Lytic	Pig fecal samples	SM3 (A)	–	Pedersen et al., 2020	MH393889	Susfortuna was identified as a new genus of bacteriophages with limited DNA sequence similarity to other known Cp phages
CPS1	Podoviridae (Picovirinae)	Lytic	Animal feces	ATCC 13124 (A)	ATCC 13124 H3^$^ ATCC 3624^$^	Ha et al., 2019	KY996523	By randomly mutating Cp strain ATCC 13124, it was discovered that the host’s capsular polysaccharides act as a receptor to phage CPS1
CPS2	Podoviridae (Picovirinae)	Lytic	Chicken feces	ATCC 13124 (A)	ATCC 13124	Ha et al., 2018	MH248069	Bacteriophage CPS2 was isolated, characterized and found to contain endolysin LysCPS2 (Supplementary Table 1) in its genome.
phiCP39O	Siphoviridae	Lytic	Broiler chicken offal washes	–		Seal et al., 2011	EU588980	The genomes of phiCP39O and phiCP26F both contained 62 potential open reading frames. Based on similarities, capsid proteins, lytic enzymes and other proteins could be predicted. Proteomic analysis was further used to identify functional proteins
phiCP26F	Siphoviridae	Lytic	Poultry feces	–		Seal et al., 2011	GQ443085	
phiCPV1	Podoviridae	Lytic	Broiler chicken intestinal contents	–	strain 46*	Volozhantsev et al., 2011	HM640230	Genomic analysis showed that phiCPV1 contains 22 open reading frames. Based on sequence similarities, 12 proteins were predicted, including proteins involved in replication of the viral DNA, its folding, production of structural components and lytic properties
phiCP24R	Podoviridae (Picovirinae)	Lytic	Raw sewage (human)	–	CP24	Morales et al., 2012	JN800508	Twenty-two genes were predicted in the genome of phiCP24R, of which ten had significant sequence similarity to proteins of known function. Three distinct genes had lytic domains, including a zinc carboxypeptidase domain that had not been previously reported in viruses
phiCPV4	Podoviridae (Picovirinae)	Lytic	Poultry waste	ATCC 3624 (A)		Volozhantsev et al., 2012	JQ729991	The genomes of the three bacteriophages (phiCPV4, phiZP2, phiCP7R) contained 26–28 open reading frames. All three bacteriophage genomes encoded a predicted N-acetylmuramoyl-L-alanine amidase and a sporulation protein
phiZP2	Podoviridae (Picovirinae)	Lytic	Poultry feces	Strain 46		Volozhantsev et al., 2012	JQ729992	
phiCP7R	Podoviridae (Picovirinae)	Lytic	Raw sewage	CP7		Volozhantsev et al., 2012	JQ729990	
phi3626	Siphoviridae	Lysogenic	–	ATCC 3626 (B)	NCTC 3110* (B)	Zimmer et al., 2002b,a	AY082070	Phi8533 and phi3626 were isolated and characterized, after which phi3626 was further analyzed Phi3626 contained fifty open reading frames. For nineteen genes a function could be assigned. These encompassed DNA-packaging proteins, structural components, a dual lysis system, a putative lysogeny switch, and proteins that are involved in replication, recombination, and modification of phage DNA as well as sporulation. The dual lysis gene cassette of phi 3626 consists of a holin gene and an endolysin gene (Supplementary Table 1)
phi8533	–	Lysogenic	–	NCTC 8533 (B)	ATCC 3628* (C)	Zimmer et al., 2002a	–	
phiSM101	Siphoviridae	Lytic^#/^ Lysogenic^@^	–	SM101 (A)		Myers et al., 2006	CP000315	The complete genome of Cp strains ATCC 13124 (gas gangrene isolate) and SM101 (enterotoxin-producing food poisoning strain) were sequenced and compared with strain 13. Considerable genomic diversity was observed between the three Cp strains, including differences in genes encoding metabolic capabilities, strain-specific extracellular polysaccharide capsule, sporulation factors, toxins, and other secreted enzymes. SM101 contained two plasmids and the complete episomal bacteriophage phiSM101
CPAS-7	Siphoviridae or Myoviridae^%^	Lytic	Poultry farms/processing plants	–	Cp 8* ATCC 13124	Miller et al., 2010 US8956628B2	–	Oral administration of the five-phage cocktail INT-401 (CPAS-7/CPAS-12/CPAS-15/CPAS-16/CPLV-42) was found to reduce necrotizing enterocolitis-associated mortality in broiler chickens infected with Cp strain C6 by 92%, and significantly improved weight gain and feed conversion ratio. The bacteriophage preparation could successfully be administered *via* the feed or water to broilers raised in a floor pen, demonstrating its utility in a commercial setting
CPAS-12	Siphoviridae or Myoviridae^%^	Lytic	Poultry farms/processing plants	–	Cp 26* ATCC 13124	Miller et al., 2010 US8956628B2	–	
CPAS-15	Siphoviridae or Myoviridae^%^	Lytic	Poultry farms/processing plants	–	Cp 8* ATCC 13124	Miller et al., 2010 US8956628B2	–	
CPAS-16	Siphoviridae or Myoviridae^%^	Lytic	Poultry farms/processing plants	–	Cp 42* ATCC 13124	Miller et al., 2010 US8956628B2	–	
CPTA-37	Siphoviridae or Myoviridae^%^	Lytic	Poultry farms/processing plants	–	Cp 27*	Miller et al., 2010 US8956628B2	–	
CPLV-42	Siphoviridae or Myoviridae^%^	Lytic	Poultry farms/processing plants	–	Cp 27* ATCC 13124	Miller et al., 2010 US8956628B2	–	
phiCJ22	Siphoviridae	Lytic	Chicken feces	strain CJ17	ATCC 13124	Bae et al., 2021	–	The administration of phiCJ22 as food supplement in a subclinical model of necrotic enteritis, induced by Cp strain CJ17, in broiler chickens effectively controlled the disease, resulting in reduced mortality, growth performance compensation, reduced numbers of Cp strains in the gut, and improved intestinal integrity. As phiCJ22 demonstrated high stability under varying pH (between 5 and 9), temperatures (up to 60°C) and drying conditions, its use in poultry farms or food processing plants was deemed feasible

*For each study the main findings are reported. If available, the Genbank (https://www.ncbi.nlm.nih.gov/genbank/) accession number of the phage genome is listed.*

*Propagation host.

*^#^Based on the fact that phiSM101 does not contain the equivalent ϕ3626 attPP’ site and exhibits minimal similarity at the nucleotide level to ϕ3626 (Myers et al., 2006).*

*^@^Based on prophage prediction (Feng et al., 2020).*

*^[$]^Inhibition zone was observed (Ha et al., 2019).*

*^%^The family was not further specified per phage (Miller et al., 2010).*

### Applications of *Clostridium perfringens*-Targeting Phages

A limited number of *in vivo* studies has investigated the use of Cp–specific phages, alone or as cocktail, to prevent or control Cp infection-associated morbidity and mortality in broiler chickens. The readout for treatment efficacy concerned parameters related to the extent of pathogen-induced intestinal mucosa damage in the animals, i.e., decreased digestion and absorption, reduced weight gain and increased feed conversion ratio (FCR), as well as mortality rates and lesion scores.

Application of the phage cocktail “INT-401” aimed to prevent or control necrotic enteritis induced in birds challenged with Cp strain C6. This cocktail—composed of five bacteriophages: CPAS-7, CPAS-12, CPAS-15, CPAS-16, and CPLV-42 (Table 1)—was chosen for its broadened host range for Cp and non-infectious nature for non–Cp strains. Orally administered INT-401 was shown to reduce necrotic enteritis-associated mortality of infected broiler chickens by 92%, and significantly improved weight gain and FCR. The bacteriophage preparation could successfully be administered *via* the feed or water to broilers raised in a floor pen, demonstrating its utility in a commercial setting. A vaccine-derived approach also showed efficacy. However, oral administration of the bacteriophage cocktail resulted in higher reduction of mortality caused by necrotic enteritis compared with alternative methods of administration of either the vaccine or the cocktail preparation (Miller et al., 2010).

The application of a single bacteriophage as food supplement was explored in a subclinical model of necrotic enteritis induced by Cp strain CJ17, harboring *net*B coding for toxin involved in necrotic enteritis, in broiler chickens. The Siphoviridae phage phiCJ22 (Table 1), isolated from a fecal sample at a chicken farm in South Korea, demonstrated broad antimicrobial activity, with 41/45 of the strains evaluated showing sensitivity (Bae et al., 2021). Bacteriophage treatment effectively controlled necrotic enteritis resulting in reduced mortality, growth performance compensation, reduced numbers of Cp strains in the gut, and improved intestinal integrity. As phiCJ22 demonstrated high stability under varying pH (between 5 and 9), temperatures (up to 60°C) and drying conditions, its use in poultry farms or food processing plants seems feasible (Bae et al., 2021). Similar beneficial results were obtained for a podovirus Cp phage used to treat necrotic enteritis induced by a *net*B-negative Cp type A strain. Cp re-isolated from the phage-treated group did not display phage resistance (Hosny et al., 2021).

Although none of the studies above observed the development of phage resistant Cp variants, Oechslin (2018) reported that the emergence of phage-resistant bacterial variants was observed in 9 out of 11 experimental studies in which various intestinal bacteria were targeted. Only one of these studies involved Gram-positive bacteria (*Enterococcus faecalis*). As such it was hypothesized that phage resistance may be a more acute problem for Gram-negative bacteria with respect to resistance selection. However, as reported in this review and Supplementary Table 2, phage resistance was potentially observed in various studies involving Cd, e.g., in the case of Cd phages phiCDHM2 (Nale et al., 2016b) and CD140 (Ramesh et al., 1999). Whether or not the intestinal environment, as suggested by Oechslin, may be more susceptible to the evolution of phage resistance as a result of its complexity remains to be investigated. From the reviewed studies on emerging phage resistance it is clear that, although at a fitness cost for the bacteria, phage resistance is not necessarily accompanied by decreased infectivity in the intestinal milieu (Oechslin, 2018).

### Phage-Derived Endolysins Against *Clostridium perfringens*

In addition to phages, phage-derived proteins have also attracted interest as novel antimicrobials. Endolysins, in particular, have gained traction in recent years due to their broad lytic activities against Gram-negative and Gram-positive bacterial cells (Love et al., 2020; Abdelrahman et al., 2021), including Cp. Other advantages of this strategy over whole phages include the absence of toxins or virulence genes and potentially a higher stability and therefore broader application range. In general, lytic enzymes are composed of two distinct domains connected *via* a linker. These include a N-terminal catalytic domain that hydrolyzes specific sites within peptidoglycan and a C-terminal cell wall-binding domain.

In 2002, the first endolysin from Cp phage phi3626 was characterized (Zimmer et al., 2002b). Since then, other phages and associated endolysins have been described, as shown in Supplementary Table 1. Virtually all endolysins originating from Cp phages exclusively showed lytic activity against Cp. A recent study on the endolysin Psa and its catalytic domain (Psa-catalytic domain) shed light on the governing factors of this specificity (Sekiya et al., 2021a,b). The species specificity of Psa lytic activity primarily depended on the binding specificity of the cell wall-binding domain, as also observed for other endolysins (Zimmer et al., 2002b). Based on X-ray and modeling studies, the Psa-catalytic domain was also found to exhibit species-specific enzymatic activity. This was apparently due to the unique Cp peptide bridges that are close to the bond that is cleaved by the endolysin. In Cp, this bridge involves the cross-linking by L,L-diaminopimelic acid, while directly cross-linked meso-diaminopimelic acid is found in most other clostridia. L,L-diaminopimelic acid is accommodated especially in binding groove-2 of the Psa-catalytic domain. This is in line with the observed lytic activity of Ply3626 toward *C. fallax*, implicated in gas gangrene. Some *C. fallax* strains share the same type of peptidoglycan (A3γ) as Cp, i.e., an L,L-diaminopimelic acid-glycine bridge to the terminal alanine of the opposite peptide chain (Zimmer et al., 2002b).

### Applications of Endolysins Targeting *Clostridium perfringens*

The applicability of Cp endolysins have so far only been studied *in vitro*. Lysins ZP173, ZP278, and Psm (Supplementary Table 1) were investigated for their utility to control food contamination (Kazanavičiūtė et al., 2018). The endolysins examined were lytic *in vitro* against various strains. Under conditions of improperly kept food, the lysins protected cooked turkey from Cp NCTC8237 spoilage. Some of the phage endolysins (Psm and ZP173) conferred better protection than nisin—the only bacteriocin approved as a food preservative. In contrast to nisin, these endolysins were able to completely eradicate the bacteria with no colonies after 18 or 43 h of incubation (Kazanavičiūtė et al., 2018). The fact that the lysins were expressed by *Nicotiana benthamiana* leaves posed an advantage over *Escherichia coli* expression when it concerns food applications. In addition to high protein yields, plant-based expression systems using Generally Recognized as Safe hosts (food species) are anticipated to be safe food additives or processing aids even if not completely purified. Therefore, production costs and process complexity may be reduced (Kazanavičiūtė et al., 2018). In addition to the lysins described by Kazanavičiūtė et al. (2018), endolysin PlyCP41 was also successfully produced in plant tissue with suggested use of crude plant sap as potential Cp antimicrobial agent (Hammond et al., 2019).

An interesting premise for increased antimicrobial activity of endolysins is the use of a “cocktail,” in analogy to phage cocktails. To our knowledge, this strategy has to date only been tested *in vitro*. In the case of Cp, the combination of endolysins Psa and Psm (Supplementary Table 1) showed a synergistic effect on the lysis of the bacterium. This was due to the fact that both endolysins target different regions in the peptidoglycans (Sekiya et al., 2021a). A synergistic killing effect was already demonstrated for two phage-encoded lysins for *Streptococcus pneumoniae*, which similarly target distinct regions in the cell wall of the host bacterium (Loeffler and Fischetti, 2003). However, the *in vivo* efficacy of such a combination approach remains to be confirmed. Suggested applications of the enzyme mixture included the spraying of areas to treat drug-resistant Cp, pre-mixing into poultry feed against foodborne Cp infections, and enteric capsule preparations for precision targeting to fight intestinal diseases (Sekiya et al., 2021a).

An alternate combination therapy showing initial promise is the expression of a biologically active anti-Cp endolysin by a probiotic bacterium that has the capability to exclude Cp from the gastrointestinal tract of poultry birds. *Lactobacillus johnsonii* FI9785, shown to reduce colonization and shedding of Cp in chickens after a single oral dose, was engineered to express CP25L (Supplementary Table 1). The nisRK two-component regulatory system from the *Lactococcus lactis* nisin A biosynthesis operon was integrated into the bacterial chromosome to allow constitutive expression of the endolysin under the control of the nisA promoter. Secretion of the active endolysin to *L. johnsonii* culture supernatants was achieved using a signal peptide (SLPmod) and subsequent lytic activity was demonstrated *in vitro* by growing colonies of *L. johnsonii* on plates incorporating autoclaved Cp cells. Zones of lysis developed around colonies expressing the lysin (Gervasi et al., 2014a). However, survival of the *L. Johnsonii* strain was shown to be cumbersome in follow-up co-culture studies, which mirrored the complex microbial communities and varying conditions of the gastrointestinal tract. It was therefore concluded that the delivery of endolysin from a species that is able to better cope with conditions in selected areas of the gastrointestinal tract might be more suitable (Gervasi et al., 2014b).

In addition to the application of wild type endolysins, fused endolysins have been investigated for introducing either improved or novel properties. These studies are described in the engineering section.

## Bacteriophages and Phage-Derived Endolysins Against *Clostridioides difficile*

### Phages Against *Clostridioides difficile*

The first study to characterize Cd-specific phages as a lead up to assessing their therapeutic merit involved four clinically relevant phages (Goh et al., 2005). These phages—isolated from patients with Cd-associated diarrhea—were found to be non-lytic and belong to the Myoviridae (phiC2, phiC5, phiC8) and Siphoviridae (phiC6) subfamilies of Caudovirales. Cd phages described before and since (Supplementary Table 2) have also been classified to these two subfamilies and are of a similar lysogenic lifestyle. The lysogenic nature of Cd phages has been suggested to be due to high integrase concentrations, favoring the lysogenic rather than the lytic cycle, as observed for phage JD032 (Li T. et al., 2020). However, several of the phages identified are not integrated in the host genome, but are episomal instead (Smits et al., 2022). To date, this has mainly been observed for large phages of around 130 kbp (i.e., phiCD5763, phiCD5774, phiCD211), although phiCD38-2 (41 kbp) is also presumed to be a plasmid prophage (Sekulovic et al., 2011).

As illustrated in Supplementary Table 2, several of the phages have broad *in vitro* activity, covering a multitude of Cd ribotypes and strains. For example, phiCDHM3 was able to lyse 31 isolates representing 12 ribotypes (Nale et al., 2016b). Another myovirus, CDKM9, was found able to infect ribotype 027, an activity generally restricted to siphoviruses (Rashid et al., 2016).

In terms of the molecular interactions between host and infecting phage, little is known. However, binding studies showed that phiHN10 (Supplementary Table 2) can specifically bind to S-layer proteins, indicating that these proteins may act as host receptors (Phothichaisri et al., 2018). From the host perspective, the conserved cell wall protein CwpV from Cd was shown to confer resistance to infection by different phages, including members of the Siphoviridae and Myoviridae families (Sekulovic et al., 2015). Cell wall protein CwpV is thought to prevent phage DNA from entering the cell, a mechanism reminiscent of superinfection exclusion systems encoded by other lysogenic phages (Sekulovic et al., 2015).

Whole genome sequencing of phiCDHM1 (Supplementary Table 2) showed that this phage contains homologs of accessory gene regulator (*agr*) genes, which make up the host quorum sensing system (Hargreaves et al., 2014). The phage *agr* genes were proposed to be of a host origin—acquired *via* horizontal gene transfer or phage infection—and either evolving within the phage genome or representing a subtype. As the genes were transcribed and retained during replication, the genes are likely of benefit in promoting the survival and replication of phiCDHM1 and its host. This may involve lysogen allelopathy (i.e., enhancing the fitness of their hosts through killing of susceptible competitors) or inhibition of secondary phage infection (Hargreaves et al., 2014).

Phage phiSemix9P1 (Supplementary Table 2), isolated from soil, has a functional binary toxin locus (CdtLoc) in its genome. Although unique to this phage, it demonstrates that lysogenic phages may play an important role in the spread of the toxin gene to pathogenic Cd strains. Similar chromosomal incorporations of virulence factors have not been observed for other clostridia (Riedel et al., 2017).

The fact that all characterized Cd phages are lysogenic poses some additional challenges compared to, for example, lytic Cp phages when considering therapeutic applications. As described in various studies, lysogenization of the Cd host may result in resistance by multiple mechanisms, such as immunity to superinfection. The lysogen may also show increased toxin production, as observed for CD274/phiCD38-2 (Supplementary Table 2). Various strategies have been explored to overcome these hurdles, including phage cocktails, phage-derived proteins, and engineering approaches.

### Applications of *Clostridioides difficile*-Targeting Phages

The efficacy of phage monotherapy has been investigated in various studies. In an *in vitro* batch fermentation model, prophylactic phiCD27 (Supplementary Table 2) treatment resulted in significantly reduced Cd cell numbers and prevented TcdA and TcdB production. Phage phiCD27 treatment after 6 h of Cd growth was less effective and resulted in re-growth of the pathogen due to lysogenization of phiCD27 with its host strain NCTC 1120 (Meader et al., 2010). Phage resistance was also observed in one of three replicate experiments during prophylactic phiCD27 therapy in a weir cascading system simulating the proximal-to-distal gut environment. In two other experiments, Cd cell numbers were significantly reduced in this *in vitro* human colon model (Meader et al., 2013). In both studies on the effectiveness of phiCD27, commensal microbiota was unaffected by the treatment.

Single-phage treatment of Cd strains CD105LC2 and CD105HE1 resulted in limited clearing of the cultures in an *in vitro* study by Nale et al. (2016b). The most effective phage (phiCDHM2; Supplementary Table 2) showed undetectable bacterial counts by 5 h, but regrowth by 24 h, which may be due to emerging phage resistance. Combining multiple phages prevented the appearance of resistant/lysogenic clones. With phage cocktails, a lytic activity on 18 of the 21 clinically relevant ribotypes tested was observed, including ribotypes 002, 005, 014/020, 015, and 078 (Nale et al., 2016b). A four-phage cocktail (phiCDHM1-phiCDHM2-phiCDHM5-phiCDHM6) was also more effective in reducing CD105LC2 biofilms *in vitro* (Nale et al., 2016a) and eradicated cultures of a clinical ribotype 014/020 strain in fermentation vessels spiked with combined fecal slurries from four healthy volunteers (Nale et al., 2018). Consistent with other studies, a prophylactic regimen was more effective at clearing Cd than a remedial regimen, where the phage cocktail was added after 5, 24, 36, and 48 h after bacterial inoculum was added. Nevertheless, Cd was fully lysed during both. Neither treatment regimens had a significant negative impact on the viability of five common major bacterial groups in the human gut (enterococci, bifidobacteria, lactobacilli, total anaerobes, and enterobacteria), concurring with previous studies by Nale using this four-phage cocktail. On the contrary, a significant abundance increase was observed in the phage-treated vessels for enterobacteria, lactobacilli, and total anaerobes, which was suggested to add to the prevention of Cd colonization (Nale et al., 2018).

In the first *in vivo* study examining single-phage treatment efficacy, phage CD140 (Supplementary Table 2) was orally administered to hamsters challenged with Cd strain 135I (Ramesh et al., 1999). One of the challenges accompanying oral administration is phage viability, as most of the phages succumb under the harsh acidic environment of the stomach. Acid sensitivity of CD140 presented an issue that was overcome by bicarbonate neutralization of gastric acid prior to phage treatment (Ramesh et al., 1999). This approach has also been applied in other studies. Although effective, dilution of phages may impact their effectiveness. In the study by Ramesh, a single phage CD140 dose at the time of Cd challenge was sufficient for all treated animals to survive, in contrast to untreated animals. However, the treated animals were not resistant against Cd re-challenge two weeks after bacteriophage treatment discontinuation. The short-term effectiveness of CD140 indicates that bacteriophages are not retained in the gastrointestinal tract. This is to be expected: if host bacteria are killed bacteriophages will disappear. In the study it was noted that one of the animals receiving multiple bacteriophage doses still developed CDI. The recovered strain was not lysed by CD140, indicating preexisting resistance or the development of resistance to CD140 (Ramesh et al., 1999).

In another *in vivo* study, phage cocktails of two or four phages were effective in reducing CD105HE1 colonization in a hamster model of CDI, as measured by bacterial counts (Nale et al., 2016b). All animals were sensitized to Cd by orally administering clindamycin 5 days prior to challenge with bacterial spores. The first treatment with single or combination phages was given at the time of Cd challenge, with subsequent doses administered every 8 h until the endpoint of 36 h, when toxin production in the cecum and colon in untreated animals was found to be limited. Subsequently, total bacterial load (spores and vegetative cells), viable counts of recovered bacteria and numbers of spores in the cecum, colon and cecal contents were determined. The reduction in levels of bacterial counts were at least 4 log units (lumen) or approximately 2 log units (tissues) for the two-phage combinations. The four-phage combination (phiCDHM1-phiCDHM2-phiCDHM5-phiCDHM6; Supplementary Table 2) showed similar results. Despite reduced colonization, Cd was still detectable in the cecum and colon of most animals. However, analysis of individual isolates from treated animals showed that the recovered bacteria were still sensitive to the phages used in the treatment. It was therefore suggested that either the multiplicity of infection was too low, or the bacteria were physiological insensitive to infection of the phage encountered. In addition to colonization, phage treatment also affected sporulation. Especially in the colon, the number of spores recovered in treated animals was substantially lower than in controls, dropping 2 log units. For analysis of disease onset involving CD105HE1, treatment with phages at the time of Cd challenge was continued every 8 h until the experimental endpoint (i.e., a core body temperature drop to 35°C at which recovery is no longer possible). The four-phage treatment delayed the CDI-associated core body temperature drop on average with 33 h compared to untreated hamsters (Nale et al., 2016b). Thus, phage cocktail treatment could delay—but not prevent—irreversible disease onset. The effectiveness of the four-phage cocktail was also compared to monotherapy in a *Galleria mellonella* larva CDI model induced with strain CD105LC2 (Nale et al., 2016a). Again, the cocktail was more effective in killing the pathogen. As observed in other studies, prophylactic treatment showed better results than remedial treatment. In the latter set-up, phage treatment was successful as an adjunct to the first-line antibiotic vancomycin (Nale et al., 2016a).

Moving toward a more human translatable model of phage intervention, Shan et al. (2018) studied the *in situ* dynamics between phiCDHS1 (Supplementary Table 2), *C. difficile* CD105LC1 (ribotype 076) and HT-29 cells. The choice of HT-29 cells for simulating colonic CDI was based on the pathogen’s ability to adhere to this human colon tumorigenic cell line (Drudy et al., 2001) and the cell line’s ability to withstand anaerobic conditions (Shan et al., 2018). The study showed that free Cd numbers were more effectively reduced in the presence than absence of HT-29 cells by phiCDHS1. Adhered Cd was also significantly reduced compared to non-phage-infected controls, whereas phage production was increased in the three-component system of phage, pathogen, and human colonic cells. No cytotoxic effect of the phage on HT-29 cells was observed, indicating that toxin release was not occurring in this study. However, the timing of intervention may be key in this respect. To prevent toxin release, Cd cells need to be killed before they can reach the toxin-producing stationary phase (Hofmann et al., 2018). As both Cd (60%) and phiCDHS1 (70%) were found to adhere to HT-29 cells, it was proposed that the increased phage activity in the presence of the human cells is due to facilitated interaction between the two entities on the HT-29 monolayer. Phage phiCDHM6 (40%), but not phiCDHM3, also adhered to the human cell line (Shan et al., 2018). The observation that phages colonize the gut and protect it against bacterial infections supports the benefits of prophylactic use observed in other studies.

### Phage-Derived Endolysins Targeting *Clostridioides difficile*

To overcome the emergence of lysogens and the potential release of toxins, the application of phage-derived endolysins targeting Cd has been explored in a limited number of studies (Table 2). Especially the benefits of using a catalytic domain-only protein has been a major focus of research.

**TABLE 2:** Endolysins targeting *C. difficile* characterized to date, as described in the referenced literature.

Endolysin	Enzymatic activity	Phage	Parent strain (Ribotype)	Cd sensitive strains (Ribotype)	Study references	NCBI reference sequence	Main findings of the study
CD27L	N-acetylmuramoyl-L-alanine amidase (amidase_3 domain; Zn-dependent)	phiCD27	NCTC 12727	strain 11204^#^ NTC11204–NTC11209 NCTC 11223 NCTC 12726–NCTC 12728NCTC 12731 NCTC 11382 NCTC 13287 NCTC 13307 NCTC 13366	Mayer et al., 2008	YP_002290910.1	Endolysin CD27L was subcloned from phiCD27 and expressed in *E. coli.* The partially purified endolysin showed lytic activity against 30 Cd strains^#^, including the major epidemic ribotype 027. CD27L was inactive against other Clostridia species or examples of the major clostridial clusters commonly represented in the gastrointestinal tract (clusters III, IV, XIVa, XIVb, XV, and XVI). For envisaged gastrointestinal tract applications, expression of CD27L by a heterologous Lactococcus lactis system was established
plyCD	Amidase	–	CD630 (012)	ATCC 43255 (087) 139B 112C	Wang et al., 2015	YP_001088405.1	The catalytic domain of plyCD (PlyCD1–174) showed a broader Cd host range than the full-length structure. Besides Cd, PlyCD1–174 was only active against *B. subtilis*. When following vancomycin pre-treatment, PlyCD1–174 significantly reduced the titer of Cd *in vitro* at doses at which both interventions were ineffective as monotherapy. PlyCD1–174 demonstrated *ex vivo* efficacy against Cd vegetative cells infecting the murine large intestine in the presence of its contents. Colonization by Cd was significantly reduced compared to controls. Rectal delivery of PlyCD1–174 in a CDI mouse model was hindered by blocked colons, leading to confounding results
CDG	N-acetylmuramoyl-L-alanine amidase	–	DA00211	strain 630 (012) R20291 (027) LiV22 (106) CD196 UK1 TL194 M68 CF5 TL-176 TL-178 Liv24 (001) CD305 (023) BI-9 (001) M120 (078)	Mehta et al., 2016	EQH20562.1	Two endolysins were cloned, expressed, purified and tested for their lytic activity *in vitro*. When treated with both CD11 and CDG *in vitro*, >90% killing of Cd strain 630 was achieved within 2 h. After 5 h, >99.99% of cells were killed. The enzymes were selective for Cd, with no observed activity for *Bacillus* or *Staphylococcus* species
CD11	N-acetylmuramoyl-L-alanine amidase	–	Strain 630 (012)	strain 630 (012) R20291 (027) LiV22 (106) CD196 UK1 TL194 M68 CF5 TL-176 TL-178 Liv24 (001) CD305 (023) BI-9 (001) M120 (078)	Mehta et al., 2016	WP_009895119.1	
LCD	–	phiC2	–	R20291 (027) M120 (078) VPI 10463 (087) LC693, 1377 (012) CD630 (012)	Peng et al., 2019	NC_009231.1	The catalytic domain of LCD was fused to the functional domain of human alpha-defensin 5, resulting in the fusion protein LHD. LHD showed higher lytic activity than LCD toward strains R20291 (ribotype 027) and M120 (ribotype 078) and similar activity toward VPI 10463 (ribotype 087), LC693, 1377 (ribotype 012) and CD630 (ribotype 012). The minimum inhibitory concentration of LHD was about 2-fold lower that LCD. Oral delivery of LHD to R20291-challenged mice decreased diarrhea, abolished mortality and reduced fecal levels of Cd spores and toxin
CWH^@^	Glucosaminidase; NlpC/P60	–	–	43255 APC 1401 CD630 (012) APC 1412 28196	Mondal et al., 2021	YP_009206142.1	CWH differs from other endolysins as it possesses two catalytic domains. The full-length protein and the protein consisting only of the two catalytic domains (CWH351-656) had a similar host range, with the latter showing faster lysis. Apart from Cd strains, the constructs were also able to lyse some *Bacillus cereus* and *Clostridium scindens* strains. CWH351-656 furthermore showed low activity against *Enterococcus faecalis* and *Listeria innocua*

*For each study, the main findings are reported. When available, the NCBI reference sequence (https://www.ncbi.nlm.nih.gov/protein/) of the corresponding endolysin is listed.*

*^#^Not all thirty strains tested are shown, please revert to the original paper by Mayer et al. (2008) for a full overview.*

*^©^CWH contains two catalytic domains: glucosaminidase and NlpC/P60 (Mondal et al., 2021).*

Bacteria produce a variety of particles that resemble phage tails and possess antibacterial activity despite the absence of a capsid and nucleic acid. The use of these phage tail-like particles (PTLPs) has attracted attention as an alternate means of combating Cd (Sangster et al., 2015). PTLPs have been identified in both Gram-positive and -negative bacteria and are potentially remnants of lysogenic phages. Several Cd strains produce PTLPs upon induction of the SOS response, thereby killing competing, non-self Cd strains (Gebhart et al., 2012). The homology of PTLPs to phage tail proteins may open avenues to rational design of such bacteriocins in the future. However, for the purpose of this review, the focus will be on the application of endolysins. The current status of PTLPs has recently been reviewed by Dams et al. (2019) and Heuler et al. (2021).

### Applications of Endolysins Targeting *Clostridioides difficile*

The first endolysin to be characterized was phiCD27-derived CD27L (Table 2). The enzyme was successfully overexpressed in *E. coli* and showed broad activity: all 30 Cd strains tested were sensitive, including two strains of the hypervirulent ribotype 027. Non-Cd strains, including some commensal gastrointestinal bacteria, were insensitive to the enzyme, indicating that CD27L is not expected to disturb the gut microbiota. For envisaged gastrointestinal applications, expression of CD27L by a heterologous *L. lactis* system was established (Mayer et al., 2008). Despite the observed lytic specificity in the first study, CD27L later was found able to lyse various less closely related species, i.e., *Bacillus amyloliquefaciens, Bacillus cereus*, and *Bacillus subtilis* (Mayer et al., 2011). Notably, all sensitive species shared the same peptidoglycan type A1γ. Truncation of the endolysin to its mere N-terminal catalytic domain (CD27L_1–179_), enhanced lytic activity and somewhat broadened the host range compared to the full-length enzyme. However, specificity toward peptidoglycan type A1γ species remained and was attributed to the configuration of the substrate binding site (Mayer et al., 2011).

The PlyCD catalytic domain (PlyCD_1–174_) similarly possessed superior lytic activity, with a wider Cd strain spectrum than the full-length structure (Table 2). PlyCD_1–174_ was not active against other potential intestinal Firmicutes, except *B. subtilis*. When following vancomycin pre-treatment, PlyCD_1–174_ significantly reduced the titer of Cd *in vitro* at doses at which both interventions were ineffective as monotherapy. The catalytic domain of PlyCD also demonstrated *ex vivo* efficacy against Cd vegetative cells infecting the murine large intestine in the presence of its contents. Colonization by Cd was significantly reduced compared to controls. Rectal delivery of PlyCD_1–174_ *via* an enema to test its efficacy in an *in vivo* CDI mouse model was hindered by blocked colons, leading to confounding results (Wang et al., 2015).

To circumvent difficult culturing conditions inherent to anaerobic pathogens, Fujimoto et al. (2020) developed a metagenome analysis pipeline for discovering novel prophages from overlapping bacterial and viral genomes found in the gut of 101 healthy individuals. As a proof of concept focusing on Cd, 10 open reading frames homologous to known endolysins were identified from prophages from the healthy individuals as well as 17 clinical isolates. The endolysins were subsequently all shown to be effective *in vitro* against strain ATCC 43255. Tested endolysins also demonstrated *in vivo* activity in a murine CDI model (Fujimoto et al., 2020).

Recently, an endolysin (CWH) from phiMMP01 was characterized that differs from other published endolysins and contains two catalytic domains (Table 2). Truncation studies showed that the full-length protein and the protein consisting of the two catalytic domains (CWH_351–656_) had a similar host range, with the latter showing faster lysis. Apart from Cd (Table 2), the constructs were also able to lyse some *B. cereus* and *Clostridium scindens* strains. CWH_351–656_ additionally showed low activity against *E. faecalis* and *Listeria innocua* (Mondal et al., 2021).

The studies conducted so far concur in a higher lytic activity and broader host range for truncated endolysins consisting of only the catalytic domain(s). This can be explained by the autoinhibitory role that has been attributed to the cell wall-binding domain, obscuring the catalytic site in absence of a cognate cell wall (Low et al., 2005). This presumed allosteric inhibition is lifted upon binding of the cell wall-binding domain to the pathogen cell wall. Alternatively, structures on the outer surface of the Cd cell (e.g., S-layer proteins) or other non-steric cell wall factors (e.g., charge complementarity) could simply hinder peptidoglycan accessibility to exogenously added full-length lysin (Wang et al., 2015).

## Engineering of *Clostridioides difficile* and *Clostridium perfringens* Phages and Their Endolysins

The exponentially growing molecular and pharmacological knowledge of phages and endolysins has instigated a substantial number of studies on the manipulation (Love et al., 2020; Gibb et al., 2021), as well as prediction (Boeckaerts et al., 2021), of their properties. Studies on *E. coli* phages, which have been investigated the most, have given valuable insights which lend themselves for extrapolation to other bacteriophages. Phage engineering approaches have involved natural evolution (Yehl et al., 2019) and genetic engineering—including recombination (Lenneman et al., 2021), rebooting (Kilcher and Loessner, 2019), domain swapping/fusion proteins (São-José, 2018; [Bibr B23][Bibr B23]), site-directed mutagenesis, and delivery of specific payloads (Hatoum-Aslan, 2018; Peng and Chen, 2021)—represent paths taken to steer activity. In addition to affecting host range (i.e., the bacterial genera, species and strains they can lyse), multiple studies focused on improving the physicochemical features (e.g., pH and thermal stability) of phages and endolysins. Studies pertaining to Cp and Cd are described below.

### Host Range

To overcome narrow host range and bacterial resistance, various engineering strategies have been pursued for phages and their endolysins. These include random and site-directed mutagenesis, the construction of fusion proteins, and the use of endogenous CRISPR-Cas systems as a payload to circumvent lysogenization. In order to guide engineering efforts, a molecular understanding of the multiple steps involved in phage—host interactions (Bertozzi Silva et al., 2016) is needed. For example, key to the host range is the binding of the phage’s receptor binding proteins (RBPs) to host receptors. To date, information of RBPs and corresponding host receptors is limited in general (Dunne et al., 2021), and specifically for Cp and Cd targeting phages, as described below.

A random mutagenesis study of *C. perfringens* ATCC 13124 demonstrated that this strain was resistant against phage CPS1 (Table 1) when a gene, encoding UDP-glucose 4-epimerase (GalE), was mutated. In conjunction with a reduced mutant host production of capsular polysaccharides and reduced host absorption by competitive inhibition by glucosamine and galactosamine, it was concluded that capsular polysaccharides act as a receptor to CPS1 (Ha et al., 2019). However, the corresponding receptor binding protein on CPS1 was not identified.

Mutating the conserved catalytic site of an endolysin to modify its host range may negatively impact its stability and consequently its applicability as antimicrobial agent. Ritter and Hackel (2019) therefore explored the application of molecular modeling techniques combined with yeast surface display and high-throughput sequencing for identifying thermally stabilizing mutations of the LysCP2 (Supplementary Table 1) catalytic domain. Using sequence homology-guided generative models, stabilizing mutations distal from the catalytic site were predicted. Stability information of multi-mutants was used to extract stabilizing single-mutants. From the *in silico* analyses, five designed proteins, incorporating 5–6 point mutations each, were selected, produced in *E. coli* and evaluated for thermal stability and activity. Four of the five proteins displayed improved thermal stability (max. 4°C). Moreover, three of the proteins exhibited a 4- to 5-fold greater activity than the wild type LysCP2 catalytic domain following thermal treatment (42°C for 30 min) in a Cp strain ATCC 12916 cell wall degradation assay (Ritter and Hackel, 2019).

Mayer et al. (2011) demonstrated that molecular modeling-supported analysis of an endolysin active site can guide host specificity modification. By comparing the catalytic sites of Cd endolysin CD27L_1–179_ and *Listeria* endolysin PlyPSA, rational site-directed mutagenesis was applied to broaden the CD27L_1–179_ host range to include *Listeria* species (Table 2; Mayer et al., 2011).

In an attempt to modulate efficacy, the catalytic domain of LCD (Table 2), a phiC2-derived endolysin homologous to PlyCD, was fused to the functional domain of human alpha-defensin 5, resulting in the fusion protein LHD (Peng et al., 2019). Human alpha-defensin 5 had demonstrated activity against hypervirulent Cd strains by mechanisms of membrane depolarization and bacterial fragmentation specific to this pathogen (Furci et al., 2015). LHD, showed higher lytic activity than LCD toward strains R20291 (ribotype 027) and M120 (ribotype 078) and similar activity toward VPI 10463 (ribotype 087), LC693, 1377 (ribotype 012), and CD630 (ribotype 012). The minimum inhibitory concentration of LHD was about 2-fold lower that LCD. Oral delivery of LHD to R20291-challenged mice decreased diarrhea, abolished mortality and reduced fecal levels of Cd spores and toxin (Peng et al., 2019).

The lysogenic nature of Cd phages is a limiting factor in their therapeutic application. With infection potentially leading to lysogeny, phage resistance can arise through a variety of mechanisms (Bondy-Denomy et al., 2016), as reported in multiple studies. To overcome this hurdle, Selle et al. (2020) engineered phage phiCD24-2 (crPhage) to carry a lethal genome-targeting CRISPR array, redirecting the endogenous CRISPR-Cas3 activity against the bacterial chromosome. Both host range and morphology were unaffected by the genomic modification. However, the maximum reduction of the numbers of colony forming units and the suppression of Cd culture growth (24 h time point) was higher for crPhage. Nevertheless, Cd treatment with both the wildtype (wtPhage) and crPhage resulted in the occurrence of lysogens. By knocking out key lysogeny genes (Δlys), the resistance against wtPhage and crPhage could be eliminated (Selle et al., 2020). As opposed to wtPhage, mice challenged with *C. difficile* CD19 spores showed significantly reduced fecal Cd burden when treated with crPhage. However, as observed *in vitro*, Cd vegetative cells were not eradicated by phage treatment and lysogens were observed. Several of the crPhage lysogens had lost part of the inserted genome. Thus, lysogens with an intact genome-targeting CRISPR are likely unstable (Selle et al., 2020). Treatment of mice with crPhage Δlys showed lower fecal *C. difficile* burden than the wtPhage Δlys. crPhage Δlys treatment was also devoid of tissue damage in the host, which was observed for crPhage and wtPhage Δlys. Lysogeny also occurred *in vivo* for wtPhage Δlys and crPhageΔlys, potentially because other Cd prophage genes are able to functionally complement the excised genes. Further elucidation of the mechanisms of lysogeny and its impacts on host cell physiology was deemed necessary (Selle et al., 2020).

### Phenotype

Apart from host specificity and lysogeny, delivering the phage at its target at the right dose while retaining its activity has been investigated. Phage infectiousness is subject to various physical and environmental stressors. Thus, relevant considerations for phage engineering include the routes of delivery and conditions encountered, stability of the phage during storage and formulation, and properties necessary for its envisaged application (Jończyk-Matysiak et al., 2019).

Thermal stability was conferred to endolysins targeting Cp by producing chimeric proteins (Swift et al., 2015, 2019). The cell wall-binding domain derived from an endolysin of a Cp bacteriophage targeting strain Cp39 (including PlyCP10, PlyCP41, and PlyCP26F; Supplementary Table 1) was fused with the catalytic domain derived from an endolysin of a thermophilic bacteriophage of *Geobacillus* strains. All fifteen chimeric proteins examined showed higher thermostability than the native Cp endolysins, and were able to kill swine and poultry disease-associated strains of Cp (Swift et al., 2019). Enhanced thermostability is of importance as animal feed is often heat-treated during production of feed pellets.

Several engineering steps have been taken to ensure survival of orally delivered phages at low pH. In a study by Vinner et al. (2017), Cd phage CDKM9 (Supplementary Table 2) was encapsulated in a pH responsive polymer using a flow focusing glass microcapillary device. The microcapsule was designed to protect phages in their transit through the stomach, and to disintegrate in the infected colon when encountering the typical pH of 7, thereby releasing the phages. Indeed, the encapsulated phages were stable during a 3 h exposure to simulated gastric fluid at pH 2 and underwent a burst release triggered at pH 7 (Vinner et al., 2017). This would technically support the oral ingestion of phage particles against CDI.

### *In silico* Prediction of Phage Host Specificity

The isolation of phages from environmental samples and the subsequent characterization of their host specificity is a time-consuming task, often accompanied by trial and error. Machine learning models have been generated to facilitate the host spectrum characterization of phages and are largely based on genomic information. For example, the web application HostPhinder predicts hosts based on similarities (co-occurring k-mers) between the query phage genome and the genomes of reference phages with known hosts (Villarroel et al., 2016). Recently, a conceptual framework was described for the construction of digital phagograms, a 3-layered modeling approach that can potentially predict whether phage—bacteria interactions result in productive infections. In this machine learning approach, the phage infection process is split into multiple consecutive steps that reflect the underlying microbiology at the species level. The first layer captures host recognition and adsorption *via* bacterial surface receptors, e.g., by using data regarding RBPs. The second layer covers the evolutionary dynamics between host and phage, e.g., genetic defense mechanisms such as CRISPR-Cas. The last layer incorporates metabolic conversion of the host by the virus, e.g., by using transcriptomics data (Lood et al., 2022).

A model described by Boeckaerts et al. (2021) focused on clinically relevant pathogens of the ESKAPE group. This group includes *C. difficile*, although it represented only 2% of the data set. The latter consisted of 1170 phage RBPs with known hosts. By transforming RBP calculated properties (e.g., protein sequence, DNA composition, structural features) into a numeric vector, machine learning models were trained to predict phage-pathogen infection ability. Notably, it was observed that highly dissimilar RBPs could still target the same host. The final model outperformed BLASTp host predictions for RBPs for which the similarity to annotated RBPs in the model fell below 75% (Boeckaerts et al., 2021). BLAST constitutes the gold standard protein sequence similarity tool. Recently, the BLAST extension Phirbo was described making the tool more suitable for predicting phage—host interactions (Zielezinski et al., 2021). Increased performance was obtained by incorporating sequence similarities of both phages and bacteria to a reference data set, expressed as relatedness.

A limitation of the RBP-based (Boeckaerts et al., 2021) and other models (e.g., Villarroel et al., 2016) is that predictions are not on strain, but species level. Strain-level predictions are currently hindered by lack of data but appear feasible in the near future giving the attention phages are currently fostering. A model that does predict phage—pathogenic bacterial strain interactions is one described by Leite et al. (2019). However, Clostridiaceae (0.3%) were underrepresented in this model, which was based on primary protein structure information and domain-domain interaction scores (Leite et al., 2019). Therefore, the phages and pathogens used for the training of a model, translating to an applicability domain, should be considered when applying *in silico* models.

A limitation inherent to all current *in silico* models is that not all aspects of host specificity are represented in the models. Factors, such as immunity to CRIPSR-Cas systems, superinfection resistance, host receptors etc., are not accounted for. Another limitation of models is the availability of data regarding strains to which phages are inactive, despite being an essential part of model training sets. Although encountered in some of the *in vitro* studies reviewed here, such data is generally difficult to obtain.

## Clinical Considerations and Challenges for the Therapeutic Use of Bacteriophages

While phage therapy has great potential, several aspects need to be further investigated before it can be applied in the medical practice. Firstly, a deeper understanding of the fundamental nature of phages and their working mechanisms is necessary. For example, the interaction between phages and their bacterial hosts needs to be better understood, and possible perturbations on non-target members of the microbiota need to be investigated (Divya Ganeshan and Hosseinidoust, 2019). In this sense, it is important to verify that the viral DNA does not integrate into the genome of the target bacteria or any other member of the microbiota. Additionally, it needs to be determined whether phage therapy may cause selective pressure on target and non-target species, thereby altering the balance in the microbiota (Divya Ganeshan and Hosseinidoust, 2019). Different *in vitro* and *in vivo* models (for example, mouse models) offer the possibility to conduct these studies, and some have already shown that, in a complex community, phages also impact non-target species through cascading effects (Hsu et al., 2019). Secondly, phages, unlike molecules such as antibiotics, are dynamic biological entities that can mutate and, in fact, they co-evolve with bacteria (Koskella and Brockhurst, 2014). Bacteriophages are involved in horizontal gene transfer, resulting in the bacterial host quickly acquiring (or losing) novel genes and thus biochemical properties after phage infection (Villa et al., 2019; Meaden and Fineran, 2021). These aspects create difficulties for the development of bacteriophage-based drugs, their production and application, as stability is an important requirement for therapeutic products (International Council for Harmonisation of Technical Requirements for Pharmaceuticals for Human Use [ICH], 2003). Therefore, phage-derived proteins such as lysins may be easier to develop and to apply as antibacterial products. Moreover, phage lytic enzymes may also prove to have other advantages, such as a reduced risk for the development of resistance (Roach and Donovan, 2015). Immunogenicity of endolysins is recognized as a potential issue with regard to systemic application of these lysins (Vázquez et al., 2018; Abdelrahman et al., 2021; Schmelcher and Loessner, 2021). The enzyme provokes an immune response when used systematically, resulting in the loss of its lytic activity *in vivo*. However, serious adverse effects of host immune responses to endolysins have not been observed (Vázquez et al., 2018). Although safety evaluations and immunogenicity studies to date largely support the idea of endolysins as a potential therapeutic, more research in this area needs to be carried out to ensure that different classes of lysins do not pose a significant risk to the human or animal host (Murray et al., 2021).

Reliable data from randomized clinical trials is fundamental to the validation of phage therapy. Results from a number of ongoing studies are awaited in the coming years. However, the specificity and evolvability of phages, the very same characteristics that make them promising as therapeutic agents and potentially suitable for personalized applications, may also pose challenges for the design and performance of randomized clinical trials (Pirnay et al., 2019; Brives and Pourraz, 2020). It is therefore important to consider whether specific guidelines and standards need to be adopted when testing phage efficacy in a clinical setting. It is encouraging though that the first intravenously administrated investigational phage therapy was devoid of adverse effects and infusion was well tolerated in patients suffering from severe sepsis (Petrovic Fabijan et al., 2020).

Aside from the limitations discussed above, related to our knowledge of the working mechanisms of phages and to the necessity of producing reliable data through clinical trials, the main obstacles to the application of phage therapy are of a regulatory nature (Brives and Pourraz, 2020). Phages are currently considered medicinal products in Europe, or drugs in the United States. The European Directive 2001/83/EC therefore applies to phages as “medicinal products for human use intended to be placed on the market in Member States and either prepared industrially or manufactured by a method involving an industrial process” (European Commission, 2001). This entails that (i) the efficacy and safety of phages need to be demonstrated in clinical trials, (ii) phages need to be produced in an industrial setting, or in any case according to good manufacturing practices, and (iii) there needs to be marketing authorization. Currently, phages do not fulfill these requirements: marketing authorization is absent, clinical trials are ongoing but are, to date, mostly inconclusive, and industrial production faces several challenges, including isolating, characterizing, and purifying phages, turning them into stable products, and scaling up production. The clinical applications of phages are made possible by Article 37 of the Helsinki Declaration, that allows physicians, on the basis of expert advice, to “use an unproven intervention if in the physician’s judgement it offers hope of saving life, re-establishing health or alleviating suffering” (World Medical Association, 2013). This means that, currently, the use of phages in a clinical setting in the western world is limited to compassionate care (McCallin et al., 2019). This often only becomes relevant when other treatment options have failed, and the physician in charge of the patients sends a bacterial sample to research laboratories in an attempt to identify an active phage. At this stage, the limiting factor seems to be the availability of phages specific for the desired target bacteria, so that multiple research centers may need to be involved, multiple phage banks need to be screened, or new phages need to be isolated from the environment (McCallin et al., 2019).

To bring phage therapy in routine practice several obstacles need to be taken. A change in the current drug development model is required, including its infrastructure of regulations, guidelines, and administration of proof of their efficacy in randomized controlled trials (Brives and Pourraz, 2020).

## Conclusion

The scientific work performed on bacteriophages demonstrates the value—and therefore fosters widespread interest in—these antibacterial viruses as acceptable alternatives for antibiotics, specifically if antibiotic therapies fail. Phage applications in a commercial setting seem possible in the near future. Currently, there are several companies involved in the commercialization of phages and phage-derived products. For severe intestinal diseases caused by Cp or Cd, alternative curative strategies are required in addition to—or as alternative for—antibiotics and/or vaccines. Examples described in this review highlight the potential success of phage therapies for these clostridia: these antibacterial agents are highly species- or strain-specific and are therefore far less detrimental to the intestinal microbiota. Apart from the phage itself, phage endolysins also show great potential. These lytic enzymes display high species specificity and were shown to be effective in synergy with antibiotic treatment. The observed cross-reactivity of clostridial endolysins with some *Bacillus* species would only have a limited impact on the intestinal environment where *Bacillus* species only play a marginal role.

Limitations that need to be considered when applying phage and phage endolysin therapy include phage stability in the gastrointestinal tract, influence on gut microbiota structure/function, phage resistance development, limited host range for specific pathogenic strains, phage involvement in horizontal gene transfer, and for phage endolysins, endolysin resistance, safety, and immunogenicity. Phage resistance can be mitigated by using phage cocktails or a combination with antibiotics to gain synergistic effects in suppression of the clostridial target. Lysogenic phages were effective in a prophylactic regimen in clearing Cd. A significant abundance increase was observed in the phage-treated vessels for enterobacteria, lactobacilli, and total anaerobes, which was suggested to add to the prevention of Cd colonization by competition. Further enhancement of therapies, involving phages or their endolysins, are explored by engineering approaches to optimize their application potential. These efforts are currently increasingly supported by *in silico* predictions. A hurdle to overcome for successful intestinal applications of phages and endolysins is the need for a technological solution counteracting the decay of phages and/or endolysins during gastric passage. Active delivery of these therapies in the small intestine and colon needs to be supported by gastric juice resistant encapsulation. Envisaged future success of phage and endolysin therapies requires reliable clinical trial data for phage (derived) products. Well-designed clinical trials can help overcome the regulatory obstacles currently faced for phage therapy. Additional research efforts, gaining more insight in the risks, benefits and opportunities, are essential to expand potential therapeutic use of phages and endolysins in mitigating severe diseases caused by enteric clostridial pathogens.

## Author Contributions

JVe wrote the sections of the manuscript pertaining to the phages and their endolysins in terms of their characteristics and biological activity. JVo wrote sections pertaining to the bacterial hosts and their societal impact, as well as the concluding sections. VA contributed to the clinical aspects of applying phages in a therapeutic setting. All authors contributed to the design of the review study and read, modified, and approved the submitted version.

## Conflict of Interest

The authors declare that the research was conducted in the absence of any commercial or financial relationships that could be construed as a potential conflict of interest.

## Publisher’s Note

All claims expressed in this article are solely those of the authors and do not necessarily represent those of their affiliated organizations, or those of the publisher, the editors and the reviewers. Any product that may be evaluated in this article, or claim that may be made by its manufacturer, is not guaranteed or endorsed by the publisher.
