# Supplementary material for: Battling Enteropathogenic Clostridia: Phage Therapy for Clostridioides difficile and Clostridium perfringens
Source: Front Microbiol. 2022 Jun 13;13:891790. doi: 10.3389/fmicb.2022.891790 (PMC9234517; doi:10.3389/fmicb.2022.891790)
Supplement: Supplementary file 1 [file Table_1.pdf]

Supplementary Material

1 Supplementary Tables

Supplementary Table 1 Endolysins targeting *C. perfringens* characterized to date, as described in the referenced literature. For each study, the main findings are reported. All cell-lytic enzymes are highly specific to *C. perfringens*. Only in some cases (e.g., PlyCM, CP25L), other clostridia demonstrated low susceptibility, while non-clostridia were generally resistant. When available, the NCBI reference sequence (https://www.ncbi.nlm.nih.gov/protein/) of the corresponding endolysin is listed.

Endolysin	Enzymatic activity	Phage	Parent strain	Sensitive Cp strains (Toxinotype)	Study reference	NCBI reference	Main findings of the study
**Psm**	Muramidase (glycosyl hydrolase_25 domain)	phiSM101	SM101	Strain 13 (A)<br>SM101 (A)<br>ATCC13124 (A)<br>KZ211 (A)<br>NCIMB10691 (B)<br>NCIMB10662 (C)<br>NCIMB10663 (D)<br>NCIMB10748 (E)<br><br>NCTC8237 (A) #	[1]<br><br><br><br><br><br><br><br><br>[2]	YP_699978.1	Psm, encoded by the episomal phage phiSM101 was identified. Recombinant histidine-tagged Psm exhibited potent cell-lytic activity towards all five Cp toxin types, but not other clostridial species, tested [1].<br><br>Psm was successfully expressed in plants and able to lyse NCTC8237 and other food-relevant Cp strains *in vitro*. A combination of three plant-produced lysins (Psm or ZP173, ZP278 and CP25L) was able to control the broadest spectrum of Cp food-relevant strains. Under conditions of improperly kept food, Psm protected cooked turkey from Cp NCTC8237 spoilage and conferred better protection than nisin − the only bacteriocin approved as a food preservative. Psm eradicated Cp NCTC8237 with no colonies after 18 or 43 hours of incubation [2].
**Psa**	N-acetylmuramoyl-L-alanine amidase (amidase_2 domain; Zn-dependent)	-	Strain 13	HN1314<br>Strain 13 (A)<br>SM101 (A)<br>ATCC13124 (A)	[3,4]		Psa was identified and shown to specifically lyse Cp [4]. The combination of endolysins Psa and Psm showed a synergistic effect on the lysis of Cp HN1314. This was due to the fact that both endolysins target different regions in the peptidoglycans of the bacteria (H. Sekiya et al., 2021).

**Ply3626**	N-acetylmuramoyl-L-alanine amidases (amidase_3 domain)	phi3626	ATCC 3626	NCTC 3110<br>ATCC 3626	[5]	NP_612849.1	Ply3626 has an N-terminal amidase domain and a unique C-terminal portion. The latter was hypothesized to be responsible for the specific lytic range of the enzyme. All tested Cp strains (48, not further specified) were sensitive to Ply3626, whereas bacteria belonging to other genera, including clostridia, were generally not affected.
**PlyCP39O**	N-acetylmuramoyl-L-alanine amidases (amidase_3 domain)	phiCP39O	Cp39 (A)	Cp26 (A)<br>Cp39 (A)<br>Cp509 (A)<br>Cp1048<br>Cp1113 (A)<br>CpA12916<br>Cp3626<br><br>NCTC8237	[6]<br><br><br><br><br><br><br><br>[2]	YP_002265435.1	PlyCP39O and PlyCP26F were cloned, expressed and sequenced. The endolysins were identical in the C-terminal cell wall binding domain, but only 55% identical in the N-terminal catalytic domain. Both were capable of lysing both parental phage Cp host strains as well as other Cp strains. Other clostridia species were resistant to the lytic activity of both endolysins [6].<br><br>PlyCP39O and PlyCP26F were successfully expressed in plants. Both endolysins demonstrated relatively low antimicrobial activity against NCTC8237 and other food-relevant Cp (toxinotype A) strains, probably due to the suboptimal pH conditions used (pH 5.5; used to mimic food conditions) [2].
**PlyCP26F**	N-acetylmuramoyl-L-alanine amidases (amidase_3 domain)	phiCP26F	Cp26 (A)	Cp26 (A)<br>Cp39 (A)<br>Cp509 (A)<br>Cp1048<br>Cp1113 (A)<br>CpA12916<br>Cp3626<br><br>NCTC8237 (A)[#]	[6]<br><br><br><br><br><br><br><br>[2]	YP_007004008.1	
**PlyCM**	Muramidase (glycosyl hydrolase_25 domain)	-	ATCC 13124	ATCC 13124 (A)<br>ATCC 3624 (A)<br>ATCC 12915 (A)<br>ATCC 12916 (A)<br>ATCC 12917 (A)<br>ATCC 12919 (A)<br>ATCC 3626 (B)<br>ATCC 3628 (C)<br>NCTC 8346 (D)<br>ATCC 27324 (E)	[7]	YP_695420	Cp strain ATCC 13124 was computationally mined, identifying PlyCM. PlyCM was active against 23 of 25 Cp strains tested (including 15 untyped strains). Virtually no activity against other clostridial or non-clostridial species was observed.
**CP25L**	N-acetylmuramoyl-l-alanine amidase (amidase_3 domain)	vB_CpeS-CP51	5416–97	NCTC 3110<br>NCTC 8238<br>NCTC 8239<br>5146-97b<br>5810-97b<br>2144-98b<br>2118-98b<br>2536-01b<br>2551-01b<br>4519-98b<br>2151-88b<br>6081-97b	[8]	YP_008058948	*Lactobacillus johnsonii* FI9785 – which reduces Cp numbers *in vitro* - was engineered to express CP25L. Secretion in *L. johnsonii* culture supernatants and subsequent lytic activity was demonstrated *in vitro*. However, survival of the *L. Johnsonii* strain was shown to be cumbersome in follow-up co-culture studies, which mirrored the complex microbial communities and varying conditions of the GI tract [8].

				3011-98b<br>2540598b<br>F3278<br>N151/151<br>N147/155A<br>DP2<br>DP3<br>DP5<br>CH1<br>CH2<br>FD00389c<br>FD00413c<br>FD00412c<br><br>NCTC8237[#]	 <br><br><br><br><br><br><br><br><br><br><br><br><br>[2]		CP25L was successfully expressed in plants and was functional in killing Cp strain NCTC8237 and other food-relevant Cp strains *in vitro*. A combination of three plant-produced lysins (Psm or ZP173, ZP278 and CP25L) was able to control the broadest spectrum of Cp food-relevant strains. Under conditions of improperly kept food, CP25L was able to efficiently reduce Cp NCTC8237 titers and conferred better protection than nisin [2].
**LysCPAS15**	N-acetylmuramoyl-l-alanine amidase (amidase_3 domain)	CPAS-15	-	KCTC 3269<br>NCTC 8239<br>NCTC 8798	[9]	QGF20128	LysCPAS15 was identified in the genome of Cp phage CPAS-15 and subsequently expressed. Its cell-wall binding domain fused to enhanced green fluorescent protein was also expressed resulting in the protein EGFP-LysCPAS15-CBD. Both the lysin and the fusion protein displayed broader host range than the bacteriophage. In milk, the proteins demonstrated the same host lysis and specific detection activities; food matrices did not hinder activity.
**PlyCP41***	Muramidase (glycosyl hydrolase_25 domain)	Cp41	-	Cp39 (A)<br>Cp28 (A)<br>Cp37 (A)<br>Cp41 (A)<br>Cp1113 (A)<br>ATCC 12916 (A)<br>ATCC 13124 (A)<br>JGS 1073 (C)<br>JGS 1508 (C)<br>JGS 1544 (C)	[10,11]	KX884995	PlyCP41 was expressed in *E.coli* and showed lytic activity against 75 Cp strains (isolates from poultry, swine and cattle; not all shown in the table*). Non-Cp strains tested were not lysed by PlyCP41 [10].<br><br>PlyCP41 was successfully produced in plant tissue. Both the purified endolysin and plant sap containing the protein lysed Cp strain Cp39 *in vitro*. It was suggested that crude plant sap can be used as a potential Cp antimicrobial agent [11].
**PlyCP10***	Muramidase (glycosyl hydrolase_25 domain)	Cp10	-	Cp39 (A)<br>Cp28 (A)<br>Cp37 (A)<br>Cp41 (A)<br>Cp1113 (A)<br>ATCC 12916 (A)<br>ATCC 13124 (A)<br>JGS 1073 (C)<br>JGS 1508 (C)<br>JGS 1544 (C)	[10]	KX884994	PlyCP10 was expressed in *E.coli* and showed lytic activity against 75 Cp strains (isolates from poultry, swine and cattle; not all shown in the table*). Non-Cp strains tested were not lysed by PlyCP10.

**LysCPS2**	N-acetylmuramoyl-L-alanine amidase (amidase_2 domain)	CPS2	-	ATCC 13124 (A)<br>H3<br>ATCC 3624<br>FORC25	[12]	AWG96515	Bioinformatic analysis of the CPS2 genome revealed LysCPS2, which was found to be first highly thermostable endolysin isolated from a Cp-targeting phage.
**ZP278**	Muramidase (glycosyl hydrolase_25 domain)	CPE str.4969, prophage region	-	NCTC8237 (A)[#]	[2]	WP_003469445	ZP173 and ZP278 were successfully expressed in plants and were able to lyse NCTC8237 and other food-relevant Cp (toxinotype A) strains *in vitro*. A combination of three plant-produced lysins (Psm or ZP173, ZP278 and CP25L) was able to control the broadest spectrum of Cp food-relevant strains. Under conditions of improperly kept food, ZP173 protected cooked turkey from Cp NCTC8237 spoilage and conferred better protection than nisin − the only bacteriocin approved as a food preservative. Both ZP173 and nisin furthermore effectively prevented proliferation of NCTC8237 in contaminated food up to 4 hours at higher temperatures (37 °C, 45 °C and 50 °C)
**ZP173**	Muramidase (glycosyl hydrolase_25 domain)	CPE str.4969, prophage region	-	NCTC8237 (A)[#]	[2]	WP_003469359	
**PlyCpAmi**	N-acetylmuramoyl-L-alanine amidases (amidase_3 domain)	-	ATCC 13124	Cp509 (A)<br>Cp26 (A)<br>Cp39 (A)<br>Cp776 (A)<br>Cp1036 (A)<br>Cp12916 (A)	[13]		PlyCpAmi was identified, expressed and found able to lyse various Cp strains *in vitro*.
**LysCP2**	Muramidase (glycosyl hydrolase_25 domain)	prophage	ATCC 13124	-	[14]		LysCP2 was used to validate the application of in silico modelling combined with thermal stability assays and sequencing to guide mutagenesis towards more stable endolysin variants. From these efforts, five proteins were selected, produced in E. coli and tested. Four of the five proteins displayed improved thermal stability (max. 4 °C). Three of the proteins exhibited a 4- to 5-fold greater activity than the wild type LysCP2 catalytic domain following thermal treatment in a Cp strain ATCC 12916 cell wall degradation assay.

* Not all sensitive Cp strains are shown, please revert to the publication by Swift (2018) for a full overview.

# The sensitivity of 26 *C. perfringens* toxinotype A strains, isolated mostly from food or from human feces after documented cases of food poisoning, were tested in addition to Cp strain NCTC8237. However, the lytic activity of the endolysin studied was not reported for each of these Cp strains [2].

References

1. Nariya H, Miyata S, Tamai E, Sekiya H, Maki J, Okabe A. Identification and characterization of a putative endolysin encoded by episomal phage phiSM101 of *Clostridium perfringens*. Appl Microbiol Biotechnol. 2011;90(6):1973–9.

2. Kazanavičiūtė V, Misiūnas A, Gleba Y, Giritch A, Ražanskienė A. Plant-expressed bacteriophage lysins control pathogenic strains of *Clostridium perfringens*. Sci Rep. 2018 Jul;8(1):10589.

3. Sekiya H, Okada M, Tamai E, Shimamoto T, Nariya H. A putative amidase endolysin encoded by Clostridium perfringens St13 Exhibits specific lytic activity and synergizes with the muramidase endolysin Psm. Antibiot (Basel, Switzerland). 2021 Mar;10(3).

4. Sekiya H, Kamitori S, Nariya H, Matsunami R, Tamai E. Structural and biochemical characterization of the *Clostridium perfringens*-specific Zn(2+)-dependent amidase endolysin, Psa, catalytic domain. Biochem Biophys Res Commun. 2021 Oct;576:66–72.

5. Zimmer M, Vukov N, Scherer S, Loessner MJ. The murein hydrolase of the bacteriophage φ3626 dual lysis system is active against all tested *Clostridium perfringens* strains. Appl Environ Microbiol. 2002;68(11).

6. Simmons M, Donovan DM, Siragusa GR, Seal BS. Recombinant expression of two bacteriophage proteins that lyse *Clostridium perfringens* and share identical sequences in the C-terminal cell wall binding domain of the molecules but are dissimilar in their N-terminal active domains. J Agric Food Chem. 2010 Oct;58(19):10330–7.

7. Schmitz JE, Ossiprandi MC, Rumah KR, Fischetti VA. Lytic enzyme discovery through multigenomic sequence analysis in *Clostridium perfringens*. Appl Microbiol Biotechnol. 2011;89(6).

8. Gervasi T, Horn N, Wegmann U, Dugo G, Narbad A, Mayer MJ. Expression and delivery of an endolysin to combat *Clostridium perfringens*. Appl Microbiol Biotechnol. 2014 Mar;98(6):2495–505.

9. Cho J-H, Kwon J-G, O’Sullivan DJ, Ryu S, Lee J-H. Development of an endolysin enzyme and its cell wall-binding domain protein and their applications for biocontrol and rapid detection of Clostridium perfringens in food. Food Chem. 2021 May;345:128562.

10. Swift SM, Waters JJ, Rowley DT, Oakley BB, Donovan DM. Characterization of two glycosyl hydrolases, putative prophage endolysins, that target *Clostridium perfringens*. FEMS Microbiol Lett. 2018 Aug;365(16).

11. Hammond RW, Swift SM, Foster-Frey JA, Kovalskaya NY, Donovan DM. Optimized production of a biologically active *Clostridium*

perfringens glycosyl hydrolase phage endolysin PlyCP41 in plants using virus-based systemic expression. BMC Biotechnol. 2019 Dec;19(1):101.

12. Ha E, Son B, Ryu S. *Clostridium perfringens* virulent bacteriophage CPS2 and its thermostable endolysin LysCPS2. Viruses. 2018 May;10(5).

13. Tillman GE, Simmons M, Garrish JK, Seal BS. Expression of a *Clostridium perfringens* genome-encoded putative N-acetylmuramoyl-L-alanine amidase as a potential antimicrobial to control the bacterium. Arch Microbiol. 2013 Nov;195(10–11):675–81.

14. Ritter SC, Hackel BJ. Validation and stabilization of a prophage lysin of *Clostridium perfringens* by using yeast surface display and coevolutionary models. Appl Environ Microbiol. 2019 May;85(10).
