# Supplementary material for: Battling Enteropathogenic Clostridia: Phage Therapy for Clostridioides difficile and Clostridium perfringens
Source: Front Microbiol. 2022 Jun 13;13:891790. doi: 10.3389/fmicb.2022.891790 (PMC9234517; doi:10.3389/fmicb.2022.891790)
Supplement: Supplementary file 2 [file Table_2.pdf]

Supplementary Material

1 Supplementary Tables

Supplementary Table 2 *C. difficile* phages characterized to date, as described in the referenced literature. The origin of Cd sensitive strains is indicated in italic when not originating from human. For each study the main findings are reported. If available, the Genbank (https://www.ncbi.nlm.nih.gov/genbank/) accession number of the phage genome is listed.

Phage	Family	Growth cycle	Origin	Induced Host (Ribotype)	Propagation Strain (Ribotype)	Cd sensitive strains (Ribotype)	Study reference	Genbank Accession number	Main findings of the study
**phiSemix9P1**	Myoviridae	Lysogenic	Soil	Semix9	-	-	[1]	KX905163	The complete genome of phiSemix9P1 was determined, annotated, and analyzed. It reportedly was the first lysogenic Cd bacteriophage harboring toxin genes, i.e. a complete functional binary toxin locus (CdtLoc)
**phiC2**	Myoviridae	Lysogenic[$]	diarrhea patient	CD242	CD062 (010)	-	[2,3]	DQ466086	Three of 56 clinical Cd isolates yielded double-stranded DNA phages phiC2, phiC5, phiC6, and phiC8 upon induction. Based on superinfection characteristics, phiC6 appeared most distantly related to the other phages. Nevertheless, phiC6 shares genetic sequence similarity to phiC8, which may be due to horizontal genetic transfer or recombination with phiC8 while coinfecting CD371 [2].<br><br>Characterization of the complete genome of phiC2 showed similarities with phage CD119 as well as with phiC630-1 and phiC630-2. phiC2-related sequences were found in 84% of 37 clinical Cd isolates and typed reference strains [3].
**phiC5**	Myoviridae	Lysogenic[$]	diarrhea patient	CD578	CD062 (010)	-	[2]		
**phiC8**	Myoviridae	Lysogenic[$]	diarrhea patient	CD371	CD60	-	[2]		
**phiC6**	Siphoviridae	Lysogenic[$]	diarrhea patient	CD371	CD843	-	[2]		
**JD032**	Myoviridae	Lysogenic	Pig	TW69 (078)	TW11 (078)	-	[4]	MK473382	JD032 was isolated, characterized and the global transcriptomic changes in the hypervirulent Cd strain TW11 was analyzed during JD032 infection. This

									analysis showed that bacterial host mRNA was progressive replaced with phage transcripts. The expression of various TW11 genes was altered upon JD032 infection, including antiphage genes (e.g., CRISPR-Cas). Bacterial sporulation, adhesion, and virulence factor genes were significantly downregulated.
**phiCD119**	Myoviridae	Lysogenic$^{\$}$		CD602		-	[5]	AY855346	phiCD119 was isolated, sequenced and annotated. Its DNA sequence contained 78 open reading frames; a function could be assigned to 23 gene products. The CD119 genome contained modules for lysogeny, DNA replication and packaging, structural proteins, and host cell lysis.
**phiCD5763***	Siphoviridae	Lysogenic	Human	LIBA-5763 (012)	CD630 (012)	-	[6]		phiCD5763 was identified as a big siphovirus with a large extrachromosomal circular genome and a large capsid. Through comparative genomics other big phages were identified, including phiCD5774 and phiCD2955. Big phages have unique predicted proteins, e.g., neck and tail proteins, receptor binding proteins and ligases. Based on their gene load, complex regulation of both phage and host functions was suggested.
**phiCD5774***	Siphoviridae	Lysogenic	Human	LIBA-5774 (012)	-	-	[6]		
**phiCD2955**	Siphoviridae	Lysogenic	Human	LIBA-2955			[6]		
**phiCD211/ phiCDIF1296T***	Siphoviridae	Lysogenic	Human	DSM1296^{T} / ATCC 9689 / CD211	NA$^{\#}$	-	[7]	NC_029048	phiCD211 (131 kbp genome) shares genomic similarities with other large siphophages. Its genome contained several transposase and integrase genes suggesting past recombination events with other mobile genetic elements. phiCD211 possessed a CRISPR locus and a cas3 gene. Comparative genomic analyses revealed phiCD211-like phage clusters. Large chromosome inversions were observed in some cluster members, as well as multiple gene insertions and module exchanges.
**CDKM9**	Myoviridae	Lysogenic	Soil	-	CD105HE1 (076)	CD105HS23 (001) CD105HS25 (001)	[8]	KX228399	CDKM15 and CDKM9 were isolated and subjected to sequence analyses, showing that the phages are genetically distinct from each other.

						CDNCTC11204 (001)<br>CD105KSE3 (001)<br>F1 (001)<br>CD105HS10 (005)<br>CD105LI07 (010)<br>CD105HS33 (010)<br>CD105HS14 (010)<br>CD105HS4 (014)<br>CD105HS42 (014)<br>CD105HS39 (015)<br>CD105LC27 (014/020)<br>CD105LC278 (014/020)<br>CD105LC1 (027)<br>CD105HS8 (027)<br>CD105HS35 (031)<br>CD105KSE11 (035)<br>CD105KSE5 (035)<br>CD105HE1 (076)<br>CD106 (106)<br>CD105HS46 (106)<br>R39V106 (106)<br>CD105HS6 (220)<br>CD105HS12 (220)			Comparative genomic analysis revealed differences in the genes relating to bacterial infection. Both phages were able to infect clinically relevant ribotypes R027 and R001.
**CDKM15**	Myoviridae	Lysogenic	Soil	-	CD105HE1 (076)	CDNCTC11204 (001)<br>CD105KSE3 (001)<br>F1 (001)<br>CD105HS20 (005)<br>CD105LI07 (010)<br>CD105HS4 (014)<br>CD105HS42 (014)<br>CD105LC27 (014/020)<br>CD105LC278 (014/020)	[8]	KX228400	

						CD105LC1 (027)<br>CD105HS8 (027)<br>CD105HE1 (076)<br>CD106 (106)<br>R40V0106 (106)<br>R6106 (106)<br>CD105HS46 (106)<br>R39V106 (106)<br>CD105HS22 (220)<br>CD105HS6 (220)<br>CD105HS12 (220)			
**phiCDHM1**	Myoviridae	Lysogenic	Estuary	-	CD105HE1 (076)	AUS1036 (002)<br>LEEDS003 (003)<br>AQV (003)<br>ATJ (014/020)<br>CD105LC2 (014/020)<br>ATK (014/020)<br>TL176 (014/020)<br>CD66 (014/020)<br>ANS (014/020)<br>CD89 (015)<br>CD81 (015)<br>LEEDS018 (018)<br>AKL (023)<br>AJX (023)<br>CD106 (106)<br>LV22 (106)	[9]	NC_024144.1	To assess whether the application of phage cocktails can attenuate the transduction potential of lysogenic phages, seven Cd-targeting phages were first analyzed for their host range. Eighty Cd strains, representing 21 major epidemic and clinically severe ribotypes, were tested. The phages demonstrated complementary lytic activity. Single-phage treatment of Cd strains CD105LC2 and CD105HE1 resulted in limited clearing of the cultures in vitro. The most effective phage (phiCDHM2) showed undetectable bacterial counts by 5 h, but regrowth by 24 h, possibly due to emerging phage resistance. Combining multiple phages prevented the appearance of resistant/lysogenic clones. With phage cocktails, a lytic activity on 18/21 clinically relevant ribotypes tested was observed, including ribotypes 002, 005, 014/020, 015, and 078. A four-phage cocktail (phiCDHM1-phiCDHM2-phiCDHM5-phiCDHM6) was also more effective in reducing CD105LC2 biofilms *in vitro*. *In vivo*, phage cocktails of two or four phages were effective in reducing CD105HE1 colonization in a hamster model of CDI, as measured by bacterial counts. Despite reduced colonization, Cd was still detectable in the cecum and colon of most animals, although the recovered bacteria remained sensitive to the
**phiCDHM2**	Myoviridae	Lysogenic	Estuary	-	CD105HE1 (076)	AUS1036 (002)<br>AIL (002)<br>AIJ (002)<br>ATH (002)<br>LEEDS003 (003)<br>AQV (003)<br>2007831 (003)<br>AOO (005)<br>AKR (013)<br>ARS (013)<br>ATJ (014/020)<br>CD105LC2 (014/020)<br>ATK (014/020)<br>TL176 (014/020)<br>CD66 (014/020)<br>ANS (014/020)<br>CD89 (015)<br>CD81 (015)<br>LEEDS018 (018)	[9]		

						CD106 (106)<br>LV22 (106)<br>M322630 (127)		phages used in the treatment. Phage treatment also affected sporulation. Especially in the colon, the number of spores recovered in treated animals was substantially lower than in controls. For analysis of disease onset involving CD105HE1 challenge, the time at which the core body temperature dropped to 35°C was assessed, i.e., when recovery is no longer possible. The four-phage treatment delayed the disease onset on average with 33 h compared to untreated hamsters.
**phiCDHM3**	Myoviridae	Lysogenic	Estuary	-	CD105HE1 (076)	AUS1033 (002)<br>AUS1036 (002)<br>AIL (002)<br>AIJ (002)<br>TL178 (002)<br>ATH (002)<br>2007831 (003)<br>AKR (013)<br>ARS (013)<br>ATJ (014/020)<br>CD105LC2 (014/020)<br>ATK (014/020)<br>TL176 (014/020)<br>AUS1022 (014/020)<br>CD66 (014/020)<br>ANS (014/020)<br>ATO (015)<br>CD89 (015)<br>CD81 (015)<br>LEEDS018 (018)<br>AKL (023)<br>AJX (023)<br>LEEDS023 (023)<br>AJV (027)<br>AKM (078)<br>AKP (078)<br>ALL (078)<br>APX (087)<br>CD106 (106)<br>LV22 (106)<br>M322630 (127)	[9]	
**phiCDHM4**	Myoviridae	Lysogenic	Estuary	-	CD105HS1 (012)	2007831 (003)<br>10 (012)<br>LEEDS018 (018)<br>APT (087)	[9]	
**phiCDHM5**	Myoviridae	Lysogenic	Estuary	-	CD105HE1 (076)	AUS1036 (002)<br>AIL (002)<br>AOO (005)<br>AKR (013)<br>ARS (013)<br>ATJ (014/020)<br>CD105LC2 (014/020)<br>ATK (014/020)<br>TL176 (014/020)<br>CD66 (014/020)<br>ANS (014/020)	[9]	

						ATO (015)<br>CD89 (015)<br>CD81 (015)<br>LEEDS018 (018)<br>AKL (023)<br>AJX (023)<br>ALN (026)<br>LV22 (106)<br>M322630 (127)	
**phiCDHM6**	Myoviridae	Lysogenic	Estuary	-	CD105HE1 (076)	AIL (002)<br>AIJ (002)<br>ATH (002)<br>2007831 (003)<br>AOO (005)<br>AKR (013)<br>ARS (013)<br>ATJ (014/020)<br>CD105LC2 (014/020)<br>ATK (014/020)<br>TL176 (014/020)<br>AUS1022 (014/020)<br>CD66 (014/020)<br>ANS (014/020)<br>ATU (015)<br>ATR (015)<br>CD89 (015)<br>CD81 (015)<br>LEEDS018 (018)<br>AKL (023)<br>AJX (023)<br>M322630 (127)	[9]
**phiCDHS1**	Siphoviridae	Lysogenic	Estuary	-	CD105LC1 (027)<br>R20291 (027)	AUS1025 (001)<br>AUS1021 (001)<br>AUSCD84 (001)<br>AIP (001)<br>CD001 (001)<br>AQV (003)<br>2007831 (003)<br>AOY (005)<br>AKR (013)<br>ARS (013)<br>ALV (015)<br>LEEDS018 (018)<br>UK023 (023)<br>LEEDS023 (023)<br>AUS1032 (027)<br>AUS1024 (027)<br>CD196 (027)<br>R20291 (027)	[9]

						B19 (027)<br>AJS (027)<br>AJV (027)<br>AMJ (027)<br>AMZ (027)<br>ANB (027)<br>US027 (027)<br>2006237 (027)<br>ANO (081)<br>ANQ (081)<br>APT (087)<br>ASQ (107)			
**CD140**	-	Lysogenic			135I	135I	[10]		Phage CD140 was orally administered to hamsters challenged with Cd strain 135I. A single phage CD140 dose at the time of Cd challenge was sufficient for all treated animals to survive, in contrast to untreated animals. However, the treated animals were not resistant against Cd re-challenge two weeks after bacteriophage treatment discontinuation. The short-term effectiveness of CD140 indicated that bacteriophages are not retained in the gastrointestinal tract. One of the animals receiving multiple CD140 doses still developed CDI. The recovered strain was not lysed by CD140, indicating pre-existing or newly developed resistance to CD140
**phiCD27**	Myoviridae	Lysogenic$	-	NCTC 12727	NCTC 11204	NCTC11204<br>NCTC11205<br>NCTC11207<br>NCTC11209	[11]	NC_011398.1	phiCD27 was isolated and showed lytic activity for 4 of 27 Cd strains tested. phiCD27 showed the highest sequence similarity with phiC2, especially in areas pertaining to DNA replication and modification and cell lysis. The genome of phiCD27 contained an endolysin, which was subcloned and expressed in *E. coli* (CD27L; Table 4).
**phiCD6356**	Siphoviridae	Lysogenic$	-	DPC6356	DPC6359	-	[12]	NC_015262.1	PhiCD6356 and phiCD6365 were isolated and characterized following mitomycin C induction of 43 Cd strains. The genome sequence of phiCD6356 was found to substantially differ from other phage sequences reported at that time.
**phiCD6365**	Siphoviridae	Lysogenic$	-	DPC6365	DPC6353	-	[12]		
**phiCD630-1**	Myoviridae	Lysogenic$	-	Strain 630	-	-	[3]		Mitomycin C induction of CD630 resulted in two plaque types corresponding to two phage particles: phiCD630-1 and phiCD630-1. Both
**phiCD630-2**	Myoviridae	Lysogenic$	-	Strain 630	-	-	[3]		

									phages possess the same morphology but have slightly different head sizes. Nucleotide comparison showed similarities of both phages with phiC2. Sequence similarity with phiCD119 concerned genes involved in cell lysis, lysogeny control and DNA replication [3].
**phiMMP01**	Myoviridae	Lysogenic%	CDI patients (stool)	-	CD19	CD19<br>CD93 (012)<br>CD117 (014)<br>CD125<br>CD211 (001)<br>CD273 (037)<br>CD326 *water* (078)<br>CD384<br>CD425 (001)<br>CD426 (106)<br>CD427 (001)<br>CD429 (014)<br>CD544<br>CD511 *dog*	[13,14]		Four phiMMP phages were isolated both as free particles in stool supernatant of CDI patients, as well as in the chromosomes of the Cd strains present in the corresponding fecal samples. Given their lysogenic lifestyle, it was concluded that the phiMMP phages were spontaneously induced from Cd *in vivo*. Complete genome sequencing revealed that phiMMP02 and phiMMP04 do not contain virulence factors or toxin genes. *In vitro*, these two phages were spontaneously released after 8 hrs of incubation. In the presence of subinhibitory concentrations of antibiotics, the prophage instability was increased [13].<br><br>Host range analysis was performed for all four phiMMP phages, covering 47 Cd strains of various origins (humans, animals, the environment) and ribotypes [14].
**phiMMP02**	Myoviridae	Lysogenic%	CDI patients (stool)	CD343 (014)	CD117 (014)	CD117 (014)<br>CD426 (106)<br>CD429 (014)<br>CD481 (014) *horse*<br>CD511 *dog*<br>CD515 *meat*	[13,14]	JX145341.1	
**phiMMP03**	Myoviridae	Lysogenic%	CDI patients (stool)	CD368 (014)	CD117 (014)	CD117 (014)<br>CD505 (014) *dog*	[13,14]		
**phiMMP04**	Myoviridae	Lysogenic%	CDI patients (stool)	CD380 (002)	CD73	CD19<br>CD24 (078)<br>CD73<br>CD93 (012)<br>CD105<br>CD316 *water*<br>CD337 *sewage*<br>CD427 (001)<br>CD428 (015)<br>CD430 (002)<br>CD490 *calf*<br>CD515 *meat*	[13,14]	JX145342.1	
**phiCD38-2***	Siphoviridae	Lysogenic$	Human stool	CD38	CD274 (027)	NA[&]<br>CD73<br>CD77<br>CD105<br>CD111 (027)<br>CD192 (027)	[15,16]	HM568888.1	Eight temperate phages were characterized after mitomycin C induction of six Cd difficile isolates, including phiCD38-2 [16].

						CD274 (027)<br>CD316 *water*<br>CD337 *sewage*<br>CD383 (027)<br>CD384<br>CD398<br>CD419 (027)<br>CD420 (027)<br>CD425 (001)<br>CD426 (106)<br>CD427 (001)<br>CD430 (002)<br>CD540<br>CD475 *horse*<br>CD481 (014) *horse*<br>CD490 (002) *calf*<br>CD505 (014) *dog*<br>CD515 *meat*			phiCD38-2 was characterized and shown to stimulate toxin expression when incorporated as a prophage in Cd: up to 1.6- and 2.1-fold more TcdA and TcdB, respectively, were detected when phiCD38-2 was lysogenized into a NAP1/027 representative isolate compared to the wild-type strain. All five pathogenicity locus (PaLoc) genes were also higher in the Cd lysogen. The phage did not contain toxins or virulence factors in its own genome [15].
**phiCDKH01**	Siphoviridae	Lysogenic	Human	CD34-Sr	-	-	[17]	MN718463	phiCDKH01 was isolated, characterized and shown to only share genomic similarity with phiCD24-1. The sequences of phiCDKH01 and phiCD24-1 share 89% identity.
**phiCD24-1**	Siphoviridae	Lysogenic$	Human	CD24 (078)	-	-	[16,17]	LN681534	Eight temperate phages were characterized after mitomycin C induction of six Cd difficile isolates, including phCD24-1 [16].<br><br>The sequences of phiCDKH01 and phiCD24-1 share 89% identity [17].
**phiCD24-2**	Myoviridae	Lysogenic	Human	CD24 (078)	CD19	CD19<br>CD73<br>CD93 (012)<br>CD117 (014)<br>CD125<br>CD211 (001)<br>CD273 (037)<br>CD326 (078) *water*<br>CD384<br>CD425 (001)<br>CD427 (001)<br>CD429 (014)<br>CD544<br>CD493 (078) *calf*<br>CD511 *dog*	[14]		Prophages were induced by UV light from 58 Cd isolates originating from animals and humans. The phages were further characterized and tested for their host range. As phiCD24-1 was induced with mitomycin C, it appears that different inducing conditions may release different phages from the same isolate.
**phiCD146**	Siphoviridae	Lysogenic	Human	CD146 (027)	CD274 (027)	CD19<br>CD77<br>CD105	[14]	LN681536.1	

						CD111 (027)<br>CD192<br>CD211 (001)<br>CD274 (027)<br>CD316 *water*<br>CD337 *sewage*<br>CD383 (027)<br>CD419 (027)<br>CD420 (027)<br>CD427 (001)<br>CD430 (002)<br>CD540<br>CD475 *horse*<br>CD490 *calf*<br>CD515 *meat*		
**phiCD111**	Siphoviridae	Lysogenic	Human	CD111 (027)	CD274 (027)	CD77<br>CD125<br>CD192<br>CD211<br>CD273<br>CD274<br>CD420<br>CD425<br>CD426<br>CD427<br>CD540<br>CD475 *horse*<br>CD490 (002) *calf*<br>CD515 *meat*	[14]	LN681535.1
**phiCD526**	Myoviridae	Lysogenic	Human	CD526 (014)	CD117 (014)	CD117 (014)<br>CD316 *water*<br>CD490 (002) *calf*<br>CD505 (014) *dog*	[14]	
**phiCD52**	Myoviridae	Lysogenic	Human	CD52	CD24 (078)	CD24 (078)<br>CD73<br>CD384<br>CD398	[14]	
**phiCD481-1**	Myoviridae	Lysogenic	Horse	CD481 (014)	CD515 *meat*	CD117 (014)<br>CD118<br>CD337 *sewage*<br>CD419 (027)<br>CD515 *meat*	[14]	LN681538.1
**phiCD481-2**	Myoviridae	Lysogenic	Horse	CD481 (014)	CD515 *meat*	CD73<br>CD118<br>CD337 *sewage*<br>CD515 *meat*	[14]	
**phiCD505**	Myoviridae	Lysogenic	Dog	CD505 (014)	CD117 (014)	CD19<br>CD117 (014)<br>CD326 (078) *water*	[14]	LN681539.1

						CD481 (014) *horse*<br>CD511 *dog*			
**phiCD506**	Myoviridae	Lysogenic	Dog	CD506	CD493 (078) *calf*	CD493 (078) *calf*	[14]	LN681540.1	
**phiCD508**	Myoviridae	Lysogenic	Dog	CD508 (014)	CD117 (014)	CD117 (014)<br>CD426 (106)<br>CD430 (002)<br>CD505 (014) *dog*	[14]		
**phiHN10**	Myoviridae	Lysogenic$	Human	HN10 (017)	-	CD630 (012)<br>HR118 (017)<br>HN2 (017)<br>HN6 (017)<br>HN9 (017)<br>HN21 (017)	[18]		Twelve clinical Cd isolates were induced by mitomycin C, resulting in five phages. Host range was determined for each phage by testing 92 Cd isolates. A narrow host range was observed for all phages. phiHN10 was found to specifically bind to S-layer proteins, indicating that these proteins may act as host receptors.
**phiHN16-1**	Myoviridae	Lysogenic$	Human	HN16 (017)	-	HN21 (017)	[18]		
**phiNH16-2**	Myoviridae	Lysogenic$	Human	HN16 (017)	-	HN21 (017)	[18]		
**phiHN50**	Myoviridae	Lysogenic$	Human	HN50 (017)	-	HN21 (017)	[18]		

* Plasmidial phages

# None of 50 different C. difficile isolates from various origins and ribotypes screened were susceptible to infection by the phage particles from the lysate (Garneau et al., 2018).

$ Induced with mitomycin C

@ isolated via enrichment

% initially isolated from free particles; identical prophages could be obtained by inducing C. difficile isolates from the corresponding fecal samples except for phiMMP01 (Meessen-Pinard, Sekulovic and Fortier, 2012).

& phiCD38-2 infected 99 of the 207 isolates tested (48%), among which 79 (80%) corresponded to the NAP1/027 epidemic strain. Other ribotypes sensitive to this phage were ribotypes 022, 028, 35,37, 40 and 41 (Sekulovic, Meessen-Pinard and Fortier, 2011).

References

1. Riedel T, Wittmann J, Bunk B, Schober I, Spröer C, Gronow S, et al. A *Clostridioides difficile* bacteriophage genome encodes functional binary toxin-associated genes. J Biotechnol. 2017 May;250:23–8.

2. Goh S, Riley T V., Chang BJ. Isolation and characterization of temperate bacteriophages of *Clostridium difficile*. Appl Environ Microbiol. 2005;71(2):1079–83.

3. Goh S, Ong PF, Song KP, Rily T V., Chang BJ. The complete genome sequence of *Clostridium difficile* phage φC2 and comparisons to φCD119 and inducible prophages of CD630. Microbiology. 2007;153(3).

4. Li T, Zhang Y, Dong K, Kuo C-J, Li C, Zhu Y-Q, et al. Isolation and characterization of the novel phage JD032 and global transcriptomic response during JD032 infection of *Clostridioides difficile* ribotype 078. mSystems. 2020 May;5(3).

5. Govind R, Fralick JA, Rolfe RD. Genomic organization and molecular characterization of *Clostridium difficile* bacteriophage ΦCD119. J Bacteriol. 2006 Apr;188(7):2568–77.

6. Ramírez-Vargas G, Goh S, Rodríguez C. The novel phages phiCD5763 and phiCD2955 represent two groups of big plasmidial Siphoviridae phages of Clostridium difficile. Front Microbiol. 2018 Jan 22;9(JAN).

7. Garneau JR, Sekulovic O, Dupuy B, Soutourina O, Monot M, Fortier L-C. High prevalence and genetic diversity of large phiCD211 (phiCDIF1296T)-like prophages in *Clostridioides difficile*. Appl Environ Microbiol. 2018 Feb;84(3).

8. Rashid SJ, Barylski J, Hargreaves KR, Millard AA, Vinner GK, Clokie MRJ. Two novel myoviruses from the north of Iraq reveal insights into Clostridium difficile phage diversity and biology. Viruses. 2016 Nov 16;8(11).

9. Nale JY, Spencer J, Hargreaves KR, Buckley AM, Trzepiński P, Douce GR, et al. Bacteriophage combinations significantly reduce *Clostridium difficile* growth *in vitro* and proliferation *in vivo*. Antimicrob Agents Chemother. 2016 Feb;60(2):968–81.

10. Ramesh V, Fralick JA, Rolfe RD. Prevention of *Clostridium difficile*-induced ileocecitis with Bacteriophage. Vol. 5, Anaerobe. 1999.

11. Mayer MJ, Narbad A, Gasson MJ. Molecular characterization of a *Clostridium difficile* bacteriophage and its cloned biologically active endolysin. J Bacteriol. 2008;190(20):6734–40.

12. Horgan M, O’Sullivan O, Coffey A, Fitzgerald GF, van Sinderen D, McAuliffe O, et al. Genome analysis of the Clostridium difficile phage φCD6356, a temperate phage of the Siphoviridae family. Gene. 2010 Aug;462(1–2):34–43.

13. Meessen-Pinard M, Sekulovic O, Fortier LC. Evidence of in vivo prophage induction during clostridium difficile infection. Appl Environ Microbiol. 2012 Nov;78(21):7662–70.

14. Sekulovic O, Garneau JR, Néron A, Fortier L-C. Characterization of temperate phages infecting *Clostridium difficile* isolates of

human and animal origins. Appl Environ Microbiol. 2014 Apr;80(8):2555–63.

15. Sekulovic O, Meessen-Pinard M, Fortier LC. Prophage-stimulated toxin production in Clostridium difficile NAP1/027 lysogens. J Bacteriol. 2011 Jun;193(11):2726–34.

16. Fortier LC, Moineau S. Morphological and genetic diversity of temperate phages in Clostridium difficile. Appl Environ Microbiol. 2007;73(22):7358–66.

17. Hinc K, Kabała M, Iwanicki A, Martirosian G, Negri A, Obuchowski M. Complete genome sequence of the newly discovered temperate *Clostridioides difficile* bacteriophage phiCDKH01 of the family Siphoviridae. Arch Virol. 2021 Aug;166(8):2305–10.

18. Phothichaisri W, Ounjai P, Phetruen T, Janvilisri T, Khunrae P, Singhakaew S, et al. Characterization of bacteriophages infecting clinical isolates of *Clostridium difficile*. Front Microbiol. 2018;9:1701.
